# ALKBH5‐mediated m^6^A demethylation of TIRAP mRNA promotes radiation‐induced liver fibrosis and decreases radiosensitivity of hepatocellular carcinoma

**DOI:** 10.1002/ctm2.1198

**Published:** 2023-02-15

**Authors:** Yuhan Chen, Peitao Zhou, Yixun Deng, Xinni Cai, Mingrui Sun, Yining Sun, Dehua Wu

**Affiliations:** ^1^ Department of Radiation Oncology Nanfang Hospital, Southern Medical University Guangzhou China; ^2^ The First School of Clinical Medicine Southern Medical University Guangzhou China

**Keywords:** hepatic stellate cells, monocytes, N6‐methyladenylate methylation, radiation‐induced liver injury, radiotherapy

## Abstract

**Background:**

Radiation‐induced hepatic stellate cell (HSC) activation promotes radiation‐induced liver fibrosis (RILF), a complication for hepatocellular carcinoma (HCC) radiotherapy. The demethylase alpha‐ketoglutarate‐dependent dioxygenase alkB homolog 5 (ALKBH5) decreases N6‐methyladenylate methylation (m^6^A) modification of RNA, while its role in regulating RILF pathogenesis and HCC radiosensitivity remains unknown.

**Methods:**

Methylated RNA immunoprecipitation sequencing (MeRIP‐seq) and RNA‐sequencing (RNA‐seq) were used to screen target genes regulated by ALKBH5. HSC with altered ALKBH5 expression was used to assess irradiation‐induced HSC activation and the effect of HSC on recruitment and polarisation of monocytes. Key cytokines in medium from irradiated HSC‐educated monocytes were identified by cytokine array detection. The effects of blocking ALKBH5 and key cytokines on RILF and HCC radiosensitivity were also evaluated.

**Results:**

Radiation‐induced ALKBH5 expression in HSC mediated m^6^A demethylation of toll‐interleukin 1 receptor domain containing adaptor protein (TIRAP) mRNA and activated its downstream NF‐κB and JNK/Smad2 pathways to promote HSC activation. Additionally, ALKBH5 regulated CCL5 secretion by irradiated HSC to promote monocyte recruitment and M2 macrophage polarisation. Notably, polarised monocytes secreted CCL20 to up‐regulate ALKBH5 expression in HSC, and reduce HCC radiosensitivity by activating ALKBH5/TIRAP axis in HCC cells. ALKBH5 knockdown‐combined CCR6 (CCL20 receptor) inhibitor significantly alleviated RILF and improved HCC radiosensitivity in mice. HCC patients with high ALKBH5 and TIRAP expression were prone to radiation‐induced liver injury and poor tumour response to radiotherapy.

**Conclusions:**

Collectively, irradiation up‐regulates ALKBH5 in HSC to mediate monocyte recruitment and M2 polarisation and form positive feedback to promote RILF and reduce HCC radiosensitivity. The dual roles of ALKBH5 as a microenvironmental regulator and radiosensitisation target provide new ideas for RILF prevention and radiosensitisation of HCC.

## Graphical abstract

- ALKBH5‐mediated TIRAP mRNA m^6^A demethylation promotes irradiated HSC activation
- ALKBH5 regulates the CCL5‐CCR5 axis to promote monocyte recruitment and M2 polarisation
- Irradiated HSC educated monocyte promotes HSC activation and reduces HCC radiosensitivity by CCL20 secretion
- Blocking ALKBH5‐CCR6 axis alleviates radiation‐induced liver fibrosis and improves HCC radiosensitivity

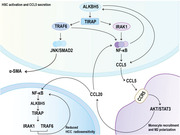

## 1 INTRODUCTION

With recent advances in radiotherapy technology, the radiotherapy effect of hepatocellular carcinoma (HCC) has been significantly improved, and radiotherapy has become one of the important treatment methods for HCC.^1^, ^2^ Although radiotherapy techniques have improved obviously, 14.7% of HCC patients still suffer from radiation‐induced liver injury (RILI) after stereotactic radiotherapy.^3^ RILI is a major complication that cannot be ignored during radiotherapy, mainly manifested as hepatitis, liver fibrosis and even death from liver failure.^4^ Radiation‐induced chronic damage to the liver is often characterised by radiation‐induced liver fibrosis (RILF).^5^ Hepatic stellate cell (HSC) is the key effector cell in the process of RILF.^6^ Radiation‐induced HSC activation is a complex process involving a variety of cytokines and pathways. Our previous studies reported that radiation induces HSC proliferation and activation through nuclear factor‐kappa B (NF‐κB) and c‐Jun N‐terminal kinase (JNK)/Smad2 pathways, which promotes RILF development.^7^, ^8^ Although it takes a certain amount of time to accumulate from liver tissue damage to liver fibrosis, there is a certain degree of liver fibrosis in the early stage of RILI.^7^, ^9^ Notably, RILF can persist for quite a long time. For example, imaging findings of RILI can still be observed in HCC patients 9 months after proton radiotherapy.^10^ Additionally, elevated expression of fibrotic factors, such as α‐smooth muscle actin (α‐SMA) and collagen 1, can be detected in a mouse model 6 months after liver irradiation.^11^ This indicates that persistent fibrosis occurs in the liver after irradiation, but the mechanism has not been reported. Although fibroblast is radiation‐tolerant, high radiation doses can still induce permanent DNA damage and irreversible cell senescence.^12^ Therefore, in addition to the activation of anti‐apoptotic and pro‐fibrotic related pathways, HSC may also act on HSC through other mechanisms in the microenvironment, thereby maintaining their fibrotic phenotype after irradiation.

Some HCC patients had obvious tumour regression or loss of activity after radiotherapy, but subsequently tumour recurrence occurred in the area of RILI, suggesting that RILI may induce changes in the liver microenvironment, leading to HCC progression or decreased radiosensitivity.^10^ HSC regulates the liver microenvironment by secreting a series of soluble factors, remodelling the extracellular matrix, promoting angiogenesis or forming an immunosuppressive microenvironment.^13^ In particular, HSC plays an important role in mediating monocyte/macrophage recruitment and polarisation to affect liver injury, liver fibrosis and HCC progression.^13^ Peripheral blood monocytes are the main source of macrophages. Macrophages can be polarised to play different functions under different conditions. M1 macrophages exhibit a pro‐inflammatory phenotype and participate in anti‐tumour immune responses. Conversely, M2 macrophages coordinate tissue repair responses and play a tumour‐promoting role.^14^ However, how irradiated HSC (IR‐HSC) regulates monocyte recruitment and polarisation as well as their impacts on RILF and HCC radiosensitivity remains unclear.

RNA is subjected to dynamic and reversible chemical modifications. The most common modification is N6‐methyladenylate methylation (m^6^A), which is dynamically regulated by a variety of RNA methyl‐transferases (‘writers’) and demethylases (‘erasers’).^15^ Alpha ketoglutarate dependent dioxygenase (AlkB) is a member of the Fe/α‐ketoglutarate‐dependent enzyme superfamily.^16^ In humans, nine AlkB homologues have been identified (ALKBH1 to ALKBH8 and FTO), which are involved in the repair of DNA and RNA through an oxidative dealkylation mechanism.^17^, ^18^ Ten‐eleven translocation 2 is also an Fe/α‐ketoglutarate‐dependent enzyme that converts 5‐methylcytosine to 5‐hydroxy‐methylcytosine on DNA.^19^, ^20^ Different from ten‐eleven translocation 2, ALKBH5 mainly takes part in m^6^A modification of RNA.^21^ As a demethylase, ALKBH5 removes m^6^A from RNA when RNA binds to the active site of ALKBH5 in a 5′‐3′ orientation and thus affects the regulation of RNA expression.^22^ The post‐transcriptional regulation of RNA mediated by ALKBH5 is crucial for pathophysiological events and has been confirmed to be involved in the occurrence and development of some diseases.^23^ This study explored the role of ALKBH5‐mediated RNA m^6^A modification in the regulation of RILF pathogenesis and HCC radiosensitivity, aiming to provide a new insight into understanding the underlying pathogenesis mechanism and the development of prevention and treatment targets.

## 2 RESULTS

### 2.1 ALKBH5 promotes IR‐HSC activation and mediates TIRAP mRNA m^6^A modification and expression

**FIGURE 1:**
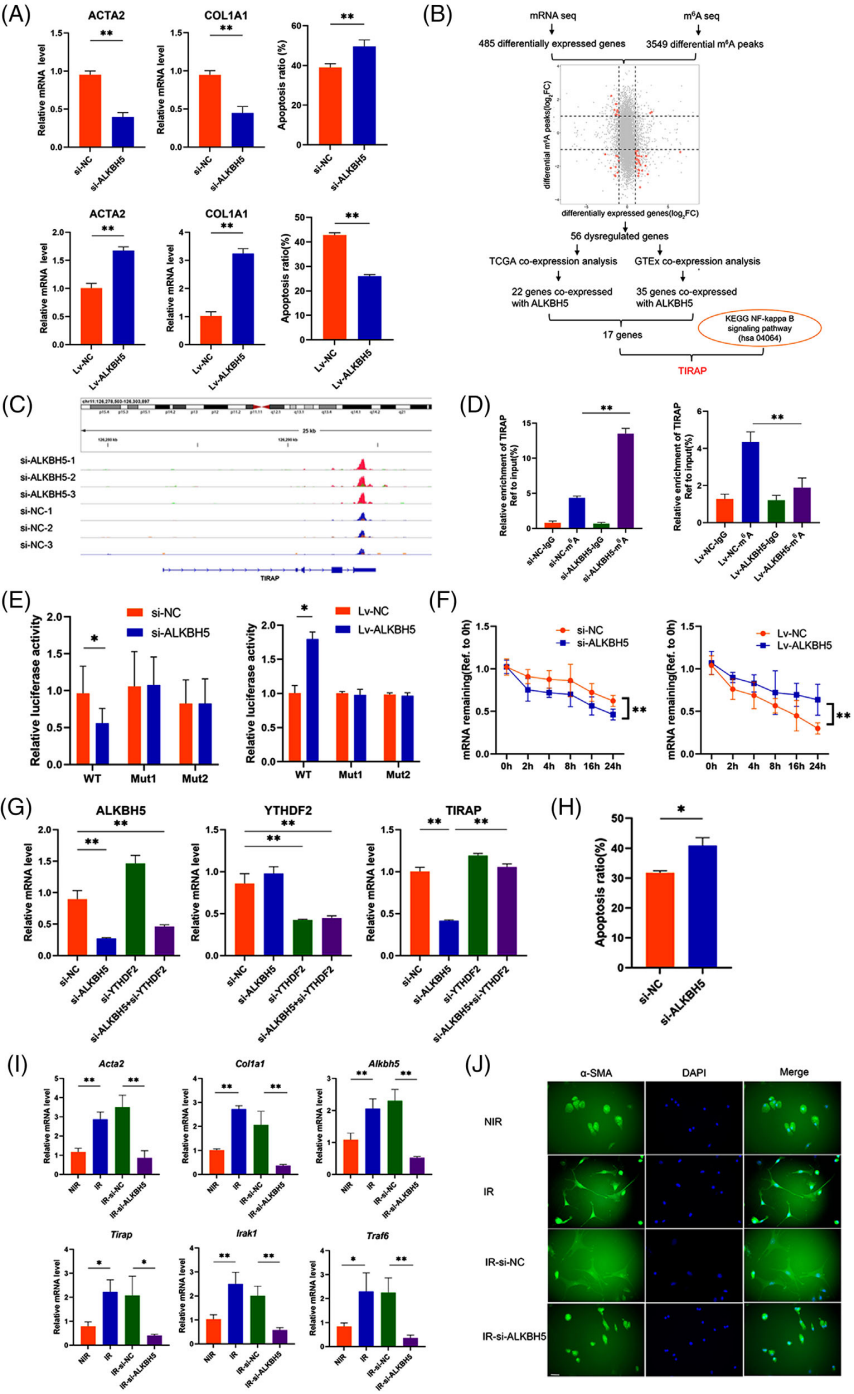
ALKBH5 mediates the N6‐methyladenylate methylation (m^6^A) modification of toll‐interleukin 1 receptor domain‐containing adaptor protein (TIRAP) mRNA. (A) mRNA level of actin alpha 2 (ACTA2) and collagen type 1 alpha 1(COL1A1) (left and middle panel) and apoptotic cell detection by flow cytometry (right panel) in irradiated LX2 (IR‐LX2) cells with knockdown or overexpression of ALKBH5. **p < .01. si‐ALKBH5, ALKBH5 siRNA; si‐NC, siRNA negative control; Lv‐ALKBH5, ALKBH5‐overexpressing lentivirus; Lv‐NC, ALKBH5‐overexpressing lentivirus negative control. (B) Flowchart of the identification of TIRAP. (C) Schematic diagram of the m^6^A abundance and modification site in the 3′‐UTR of TIRAP mRNA. (D)MeRIP quantitative real‐time polymerase chain reaction (qRT‐PCR) analyses of m^6^A modification of TIRAP in ALKBH5‐silenced or ‐overexpressed IR‐LX2 cells using anti‐immunoglobulin G (IgG) and anti‐m^6^A antibodies. **p < 0.01. (E) Luciferase activity in ALKBH5‐silenced or ‐overexpressed IR‐LX2 cells transfected with luciferase reporter containing the wild‐type (WT) (full‐length) or mutant (Mut) (m^6^A motif mutated) sequence of TIRAP–3′UTR. *p < .05. (F) RNA stability of TIRAP in ALKBH5‐silenced or ‐overexpressed in ALKBH5‐silenced or ‐overexpressed IR‐LX2 cells treated with actinomycin D and harvested at indicated time. **p < .01. (G) mRNA level of ALKBH5, YTHDF2 and TIRAP in IR‐LX2 cells with knockdown of ALKBH5 and or YTHDF2. **p < .01. (H) Apoptotic cell detection by flow cytometry of irradiated mouse primary HSC transfected with si‐NC or si‐ALKBH5. *p < .05. (I) mRNA level of fibrotic markers and Alkbh5/Tirap axis genes in non‐irradiated (NIR) mouse primary hepatic stellate cell (HSC) and irradiated (IR) mouse primary HSC transfected with si‐NC or si‐ALKBH5. **p < .01; *p < .05. (J) α‐smooth muscle actin (α‐SMA) fluorescence in mouse primary HSC with indicated treatment. Scale bar: 50 µm.

ALKBH5 was up‐regulated in irradiated LX2 cells, especially at 8 Gy dose (Figure S1A). Notably, ALKBH5 promoted irradiated LX2 activation and reduced cell apoptosis (Figures 1A and S1B). We also confirmed the conclusion in isolated primary mouse HSC (Figure 1H–J). To identify the genes regulated by ALKBH5, we performed an integrated approach combining methylated RNA immunoprecipitation‐sequencing (MeRIP‐seq) and RNA‐sequencing (RNA‐seq). The common motif of m^6^A in LX2 cells was GGACUU (Figure S1D). Compared with the control group, there were less unique m^6^A peaks (10 523 vs. 11 547) and less genes with unique modification peaks (9581 vs. 10 261) in ALKBH5 knockdown group (Figure S1E). There were 3549 differential m^6^A peaks (fold change ≥ 2, *p* < .05) and 485 differentially expressed genes (fold change ≥ 2, *p* < .05) in ALKBH5 knockdown group, and 56 genes that showed differences in m^6^A peaks and gene expression were obtained by integrated analysis. Then the co‐expression between 56 genes and ALKBH5 was verified in normal liver tissues of the TCGA database and Genotype‐Tissue Expression database, and 17 genes co‐expressed with ALKBH5 were obtained by intersection. ALKBH5 promoted the activation of NF‐κB (Figure S1C), whereas NF‐κB pathway activation is related to the proliferation and activation of HSC that promotes the development of RILI.^7^, ^8^ After intersection with the above 17 genes and NF‐κB pathway gene set from the KEGG database (https://www.kegg.jp/), only toll‐interleukin 1 receptor domain‐containing adaptor protein (TIRAP), the upstream regulatory molecule of NF‐κB, was identified to be the target gene for ALKBH5 to regulate the activation of NF‐κB pathway. Further analysis showed that the m^6^A site of TIRAP was distributed in 3' untranslated region (3′UTR) (Figure 1C). ALKBH5 promoted m^6^A modification of TIRAP and bound to the wild‐type binding sites in TIRAP 3′UTR, suggesting that ALKBH5 mediates the m^6^A modification of TIRAP mRNA (Figure 1D,E). In addition, actinomycin D assay showed that ALKBH5 improved the stability of TIRAP mRNA (Figure 1F). Previous studies have reported that m^6^A reader YTH N6 methyladenosine RNA binding protein 2 (YTHDF2) enables to recognise m^6^A and promotes the degradation of hypermethylated mRNA.^24^ Therefore, we further explored the effect of YTHDF2 on TIRAP expression. As shown in Figure 1G, YTHDF2 knockdown restored the down‐regulated expression of TIRAP caused by ALKBH5 knockdown, indicating that TIRAP mRNA was hypermethylated and then recognised and degraded by YTHDF2. These results suggest that ALKBH5 mediates the m^6^A modification of TIRAP mRNA and regulates TIRAP expression in a YTHDF2‐dependent manner.

### 2.2 TIRAP mediates the regulatory effects of ALKBH5 and has positive feedback regulation with ALKBH5

**FIGURE 2:**
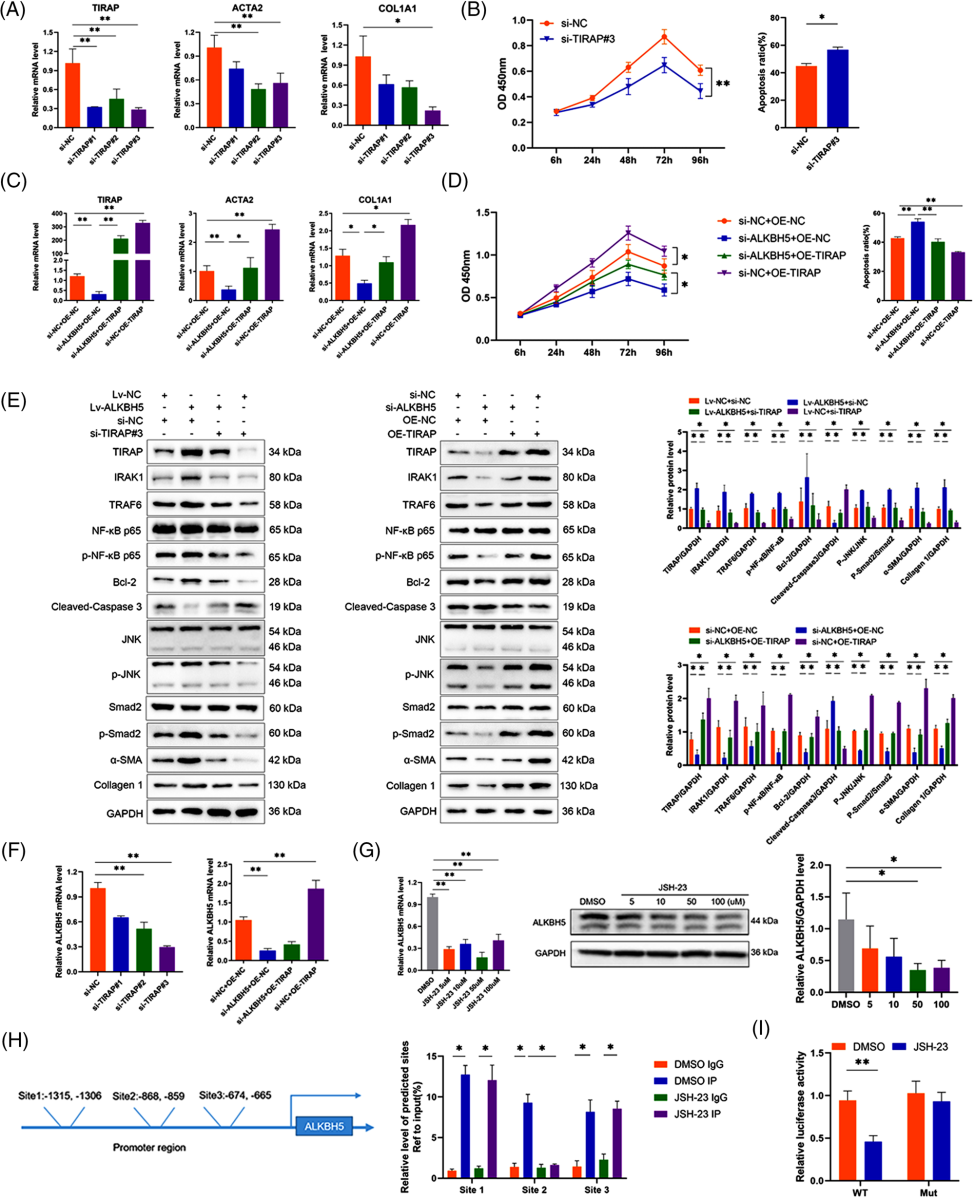
TIRAP is the target gene that mediates the regulatory effects of ALKBH5. (A) mRNA level of TIRAP, ACTA2 and COL1A1 in IR‐LX2 cells transfected with siRNA negative control (si‐NC) or different TIRAP siRNAs (si‐TIRAP). **p < .01; *p < .05. (B) The CCK8 proliferation assay (left panel) and apoptotic cell detection by flow cytometry (right panel) of IR‐LX2 cells transfected with si‐TIRAP or si‐NC. **p < .01; *p < .05. (C) mRNA level of TIRAP, ACTA2 and COL1A1 in IR‐LX2 cells transfected with si‐ALKBH5 and or TIRAP‐overexpressing plasmid (OE‐TIRAP) or negative control (OE‐NC). **p < .01; *p < .05. (D) The CCK8 proliferation assay (left panel) and apoptotic cell detection by flow cytometry (right panel) of IR‐LX2 cells transfected with si‐ALKBH5 and or OE‐TIRAP or OE‐NC. **p < .01; *p < .05. (E) Western blot analysis of the related proteins in IR‐LX2 cells with altered ALKBH5 and or TIRAP expression. *p < .05. (F) mRNA level of ALKBH5 in IR‐LX2 cells with altered ALKBH5 and or TIRAP expression. **p < .01. (G) mRNA level and western blot analysis of ALKBH5 in IR‐LX2 cells with various doses of JSH‐23 treatment. **p < .01; *p < .05. (H) The potential binding sites in ALKBH5 promoter region for NF‐κB p65 predicted by the JASPAR database. ChIP qRT‐PCR analysis of NF‐κB p65 binding sites in ALKBH5 promoter region in dimethyl sulfoxide (DMSO) or JSH‐23 (50 µM) treated IR‐LX2 cells using anti‐IgG and anti‐NF‐κB p65 antibodies. *p < .05. (I) Luciferase activity in DMSO or JSH‐23(50 µM)‐treated IR‐LX2 cells transfected with luciferase reporter containing the WT or Mut sequence of NF‐κB p65 binding site 2 in ALKBH5 promoter region. **p < .01.

Down‐regulation of TIRAP could simulate the effect of ALKBH5 knockdown, that is, inhibiting the activation and proliferation of LX2 cells and promoting apoptosis (Figure 2A,B), while TIRAP overexpression reversed the effects of ALKBH5 inhibition (Figure 2C,D). ALKBH5 upregulated the expression of TIRAP and its downstream genes interleukin 1 receptor associated kinase 1 (IRAK1) and TNF receptor associated factor 6 (TRAF6), promoted the activation of NF‐κB and JNK/Smad2 and led to up‐regulation of anti‐apoptosis factor Bcl‐2 and activation markers (α‐SMA and collagen 1), while TIRAP knockdown partially alleviated the promoting effects of ALKBH5 overexpression (Figure 2E). On the contrary, silencing ALKBH5 down‐regulated the expression of TIRAP and downstream effectors in LX2 (Figure 2E) and primary HSCs (Figure 1K), while TIRAP overexpression could reverse the inhibitory effects of ALKBH5 (Figure 2E). These results suggest that TIRAP is the target gene that mediates the regulatory effects of ALKBH5.

Interestingly, we also found that TIRAP can up‐regulate ALKBH5 expression (Figure 2F), suggesting that there is a positive feedback loop between ALKBH5 and TIRAP. It was predicted by the JASPAR database (https://jaspar.genereg.net/) that there were several binding sites in ALKBH5 promoter region for NF‐κB p65, the downstream effector of TIRAP (Figure 2H). Furthermore, we found that NF‐κB p65 inhibitor JSH‐23 suppressed ALKBH5 expression and inhibited the binding of NF‐κB p65 and binding site 2 in ALKBH5 promoter (Figure 2G–I). These results indicate that the activation of TIRAP/NF‐κB pathway promotes ALKBH5 expression, and there is a positive feedback regulation between ALKBH5 and TIRAP.

### 2.3 ALKBH5 regulates the CCL5 production of IR‐HSC

In the mouse model of RILI, the ALKBH5/TIRAP axis was significantly activated in liver tissue and HSC (Figure S2A–C). HSC activation markers α‐SMA and collagen 1 deposition were mainly distributed around the vessels (Figure S2D,E), suggesting that activated HSC is mainly distributed around the hepatic vessels after irradiation, which provides favourable conditions for HSC to participate in the recruitment of peripheral blood cells into the liver. Compared with the non‐irradiation group, more F4/80^+^ macrophages infiltrated the liver of the irradiation group (Figure S2F). CLEC4F is used to identify Kupffer cells, while IBA1 is a pan‐macrophage marker of Kupffer cells and infiltrating monocytes/macrophages. After irradiation, IBA1^+^CLEC4F^−^ monocytes were recruited around the injured hepatic sinusoids, while IBA1^+^CLEC4F^+^Kupffer cells were mainly distributed in liver tissue (Figure S2G), suggesting that irradiation promoted peripheral blood monocyte infiltration.

To test whether IR‐HSC promoted monocyte migration and polarisation, the human monocyte THP‐1 cells were co‐cultured with the culture medium of irradiated LX2 cells (IR‐LX2 CM). We found that the migration of THP‐1 was increased, the expression of M2‐related markers (CD163, CD206, IL10) was increased, while the expression of M1‐related markers (interleukin 1 beta (IL1B), tumor necrosis factor (TNF), nitric oxide synthase 2 (NOS2)) was reduced (Figure S3A–C). Similar results were observed in the co‐culture experiment of mouse IR‐HSC CM and bone marrow‐derived monocytes (BMDMs) (Figure S5A). These results suggested that IR‐HSC promoted monocyte migration and polarisation to M2. However, the ALKBH5‐silenced IR‐LX2 CM significantly reduced THP‐1 migration and polarisation to M2 (Figure S3E,F).

**FIGURE 3:**
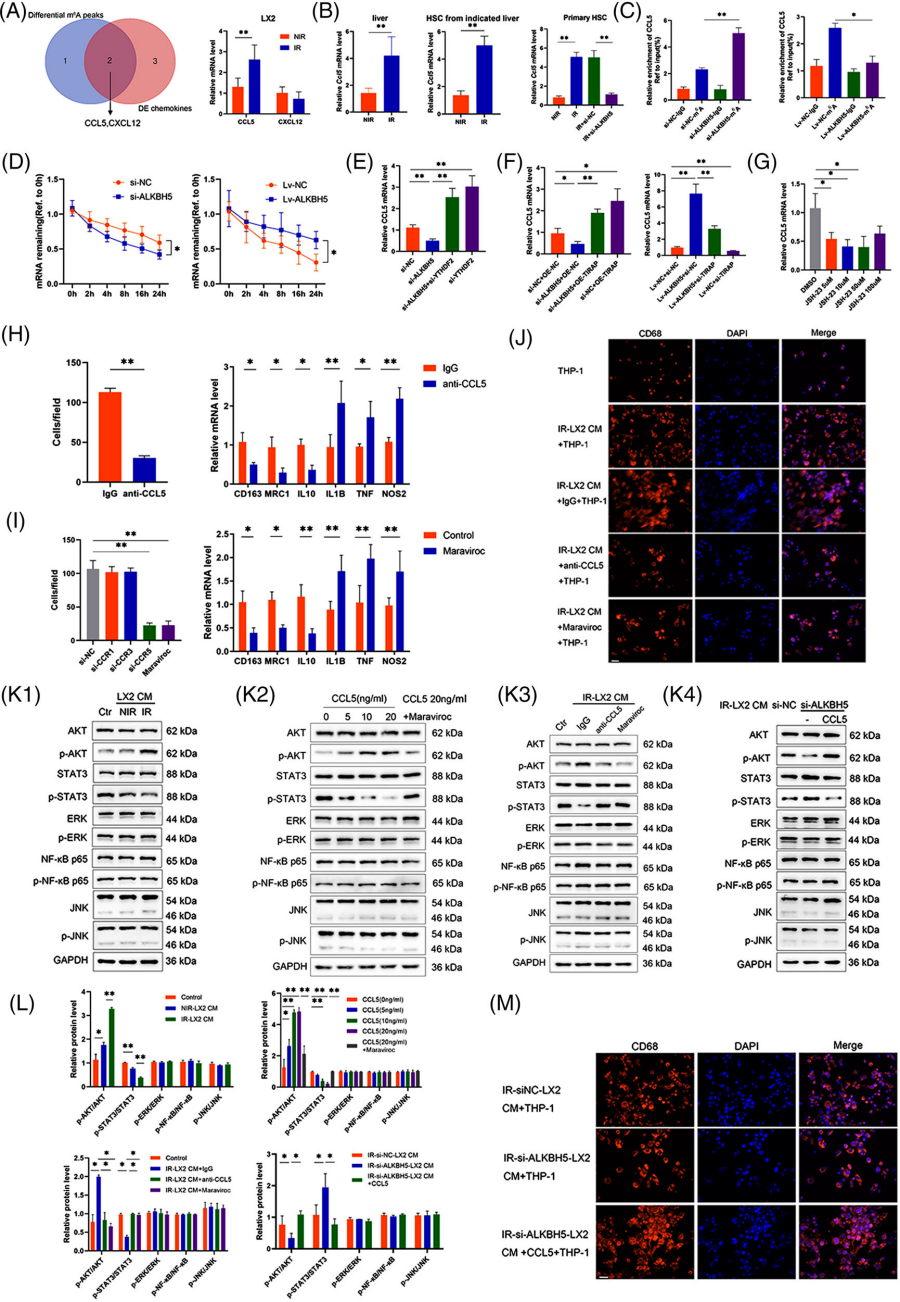
Irradiated HSC (IR‐HSC) promotes monocyte recruitment and polarisation through ALKBH5/CCL5/CCR5 axis. (A) Venn diagram for the selection of chemokines with differential m^6^A peaks and differential expression, together with verification of the mRNA level of CCL5 and CXCL2 in LX2 cells with or without irradiation. **p < .01. (B) mRNA level of *Ccl5* in liver (left panel) and HSC (middle panel) from mice with or without irradiation, together with mRNA level of *Ccl5* in primary mouse HSC (right panel) with indicated treatment. **p < .01. (C) MeRIP qRT‐PCR analysis of m^6^A modification of CCL5 in ALKBH5‐silenced or ‐overexpressed IR‐LX2 cells using anti‐IgG and anti‐m^6^A antibodies. **p < .01; *p < .05. (D) RNA stability of TIRAP in ALKBH5‐silenced or ‐overexpressed IR‐LX2 cells treated with actinomycin D and harvested at indicated time. *p < .05. (E) mRNA level of CCL5 in IR‐LX2 cells with altered ALKBH5 and or TIRAP expression. **p < .01. (F) mRNA level of CCL5 in IR‐LX2 cells with various doses of JSH‐23 treatment. **p < .01; *p < .05. (G) mRNA level of CCL5 in IR‐LX2 cells with knockdown of ALKBH5 and or YTHDF2. *p < .05. (H) Cell migration (left panel) and polarisation (right panel) of THP‐1 cells after co‐culturing with the IR‐LX2 culture medium (IR‐LX2 CM) in the presence of anti‐CCL5 neutralising antibody (10 µg/mL) or control IgG. **p < .01; *p < .05. (I) THP‐1 cells were pretreated with CCR1 siRNA, CCR3 siRNA, CCR5 siRNA or maraviroc (5 µM) and cell migration (left panel) of THP‐1 cells were detected after co‐culturing with the IR‐LX2 CM. The polarisation (right panel) of THP‐1 cells pretreated with or without maraviroc (5 µM) were detected after co‐culturing with the IR‐LX2 CM. **p < .01; *p < .05. (J) CD68 fluorescence in THP‐1 cells after co‐culturing with IR‐LX2 CM in the presence of anti‐CCL5 neutralising antibody (10 µg/mL) or maraviroc (5 µM). Scale bar: 50 µm. (K) Western blot analysis of the related proteins in THP‐1 cells with indicated treatment. (K1) THP‐1 cells co‐cultured with NIR or IR‐LX2 CM. (K2) THP‐1 cells were treated with various doses of CCL5 with or without maraviroc (5 µM). (K3) THP‐1 cells co‐cultured with IR‐LX2 CM in the presence of anti‐CCL5 neutralising antibody (10 µg/mL) or maraviroc (5 µM). (K4) THP‐1 cells co‐cultured with ALKBH5‐silenced IR‐LX2 CM with or without CCL5 (20 ng/mL) treatment. Ctr, control. (L) Relative quantification for the protein bands in (K1‐K4). **p < .01; *p < .05. (M) CD68 fluorescence in THP‐1 cells after co‐culturing with control or ALKBH5‐silenced IR‐LX2 CM with or without CCL5 (20 ng/mL) treatment. Scale bar: 50 µm.

To identify the role of ALKBH5 in monocyte infiltration and polarisation, MeRIP‐seq and RNA‐seq revealed that ALKBH5 regulates the m^6^A modification and expression of chemokines CCL5 and CXCL12 (Figure 3A). CCL5 expression was up‐regulated in IR‐LX2 cells, irradiated liver tissue and HSC of mice, but there was no significant change in CXCL12 (Figure 3A,B). Notably, CCL5 expression in isolated irradiated mouse HSC was down‐regulated by silencing ALKBH5 (Figure 3B). ALKBH5 mediated m^6^A modification of CCL5 and improved the stability of CCL5 mRNA (Figure 3C,D). Silencing YTHDF2 restored the down‐regulation of CCL5 caused by ALKBH5 knockdown (Figure 3E), indicating that ALKBH5 mediates the m^6^A modification of CCL5 mRNA and regulates CCL5 expression in a YTHDF2‐dependent manner. In addition, we also found that TIRAP can reverse the regulation of ALKBH5 on CCL5 expression (Figure 3F). Inhibition of NF‐κB activation by JSH‐23 also down‐regulated the expression of CCL5 (Figure 3G). Collectively, these results suggested that ALKBH5 also promotes CCL5 expression through the activation of TIRAP/NF‐κB pathway.

### 2.4 ALKBH5 regulates the CCL5/CCR5 axis to promote monocyte recruitment and polarisation

ALKBH5 promoted the secretion of CCL5 by HSC (Figure S3D). Adding CCL5 neutralising antibody to the co‐culture of IR‐LX2 CM and THP‐1 could inhibit the migration of THP‐1 (Figure 3H). Since the common receptors of CCL5 are CCR1, CCR3 and CCR5, THP‐1 cells were transfected with CCR1, CCR3 and CCR5 siRNA or pretreated by maraviroc (a selective inhibitor of CCR5) and then co‐cultured with IR‐LX2 CM. We found that THP‐1 migration was inhibited by CCR5 siRNA or using maraviroc but not CCR1 or CCR3 siRNA (Figure 3I). CCL5 neutralising antibody or maraviroc pretreatment reduced the morphological change and M2 polarisation of THP‐1 cells (Figure 3I,J). Similar results were observed in the co‐culture of mouse IR‐HSC CM and BMDMs (Figure S4B,C). It has been reported that the activation of AKT, signal transducer and activator of transcription (STAT), extracellular regulated protein kinase (ERK), JNK and NF‐κB pathways could be induced by CCL5/CCR5 axis.^25^ In this study, IR‐LX2 CM promoted AKT phosphorylation and inhibited STAT3 phosphorylation in THP‐1 cells but had no effects on ERK, NF‐κB and JNK phosphorylation (Figure 3K1). In addition, CCL5 promoted AKT phosphorylation and inhibited STAT3 phosphorylation in THP‐1 cells in a dose‐dependent manner, which could be reversed by maraviroc blockade (Figure 3K2). Similar results could also be observed by adding CCL5 neutralising antibody or pretreatment with maraviroc in the co‐culture of IR‐LX2 CM and THP‐1 (Figure 3K3). In order to determine whether the CCL5 secretion of HSC mediated by ALKBH5 promotes the migration and polarisation of monocytes, LX2 cells were transfected with ALKBH5 siRNA and then stimulated with CCL5. Compared with the control group, ALKBH5‐silenced IR‐LX2 CM reduced THP‐1 migration, morphological change and M2 polarisation, promoted AKT phosphorylation and inhibited STAT3 phosphorylation, which could be reversed by CCL5 stimulation (Figures S3E,F and 3K4,M). After the same treatment as above, similar results were observed in the co‐culture of mouse IR‐HSC CM and BMDMs (Figure S5D,G). On the contrary, co‐culture of ALKBH5‐overexpressing IR‐LX2 CM and THP‐1 promoted THP‐1 migration, morphological change and M2 polarisation, inhibited AKT phosphorylation but promoted STAT3 phosphorylation, which could be reversed by CCL5 neutralisation antibody or maraviroc (Figure S4A–D). In addition, when THP‐1 cells were pretreated with AKT inhibitor MK2206 and co‐cultured with IR‐LX2 CM, the migration, morphological change and M2 polarisation of THP‐1 cells decreased (Figure S4E–G). Similar results were observed in the co‐culture of mouse IR‐HSC CM and BMDMs (Figure S5E,F). Moreover, maraviroc administration in the mouse model of RILI significantly decreased the recruitment of IBA1^+^CLEC4F^−^ monocytes in perivascular tissue (Figure S2G). Maraviroc treatment also significantly reduced the level of Ly6C^+^ monocyte and decreased the level of M2 marker CD163 more significantly than that of M1 marker CD86 (reduced 36.83% vs. 7.62%; Figure S2H), suggesting that blocking CCR5 predominantly inhibits the monocyte recruitment and M2 polarisation.

**FIGURE 4:**
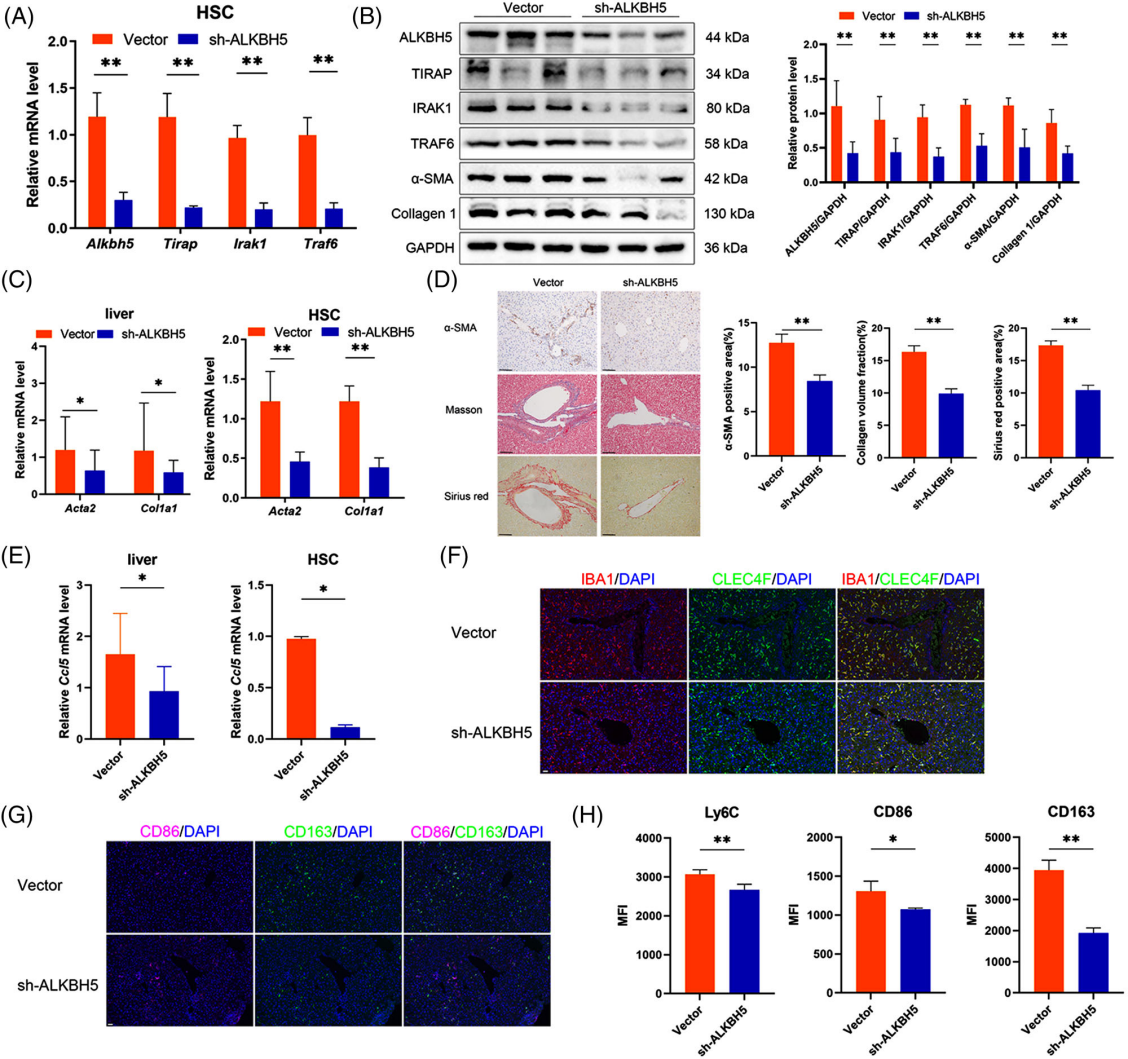
ALKBH5 inhibition in HSC alleviates radiation‐induced liver fibrosis (RILF) and monocyte infiltration. (A) mRNA level of *Alkbh5, Tirap, Irak1* and *Traf6* in HSC from mice treated with HBAAV‐GFAP‐shALKBH5 or control vector. sh‐ALKBH5, HBAAV‐GFAP‐shALKBH5. **p < .01. (B) Western blot analysis of the related proteins in HSC from mice treated with HBAAV‐GFAP‐shALKBH5 or control vector. **p < .01. (C) mRNA level of Acta2 and Col1a1 in liver and HSC from mice treated with HBAAV‐GFAP‐shALKBH5 or control vector. **p < .01; *p < .05. (D) Immunohistochemistry (IHC) staining of α‐SMA, Masson staining and Sirius red staining in liver from mice treated with HBAAV‐GFAP‐shALKBH5 or control vector. **p < .01. (E) mRNA level of Ccl5 in liver and HSC from mice treated with HBAAV‐GFAP‐shALKBH5 or control vector. *p < .05. (F) IBA1 and CLEC4F fluorescence in liver from mice treated with HBAAV‐GFAP‐shALKBH5 or control vector. Scale bar: 20 µm. (G) CD86 and CD163 fluorescence in liver from mice treated with HBAAV‐GFAP‐shALKBH5 or control vector. Scale bar: 20 µm. (H) Flow cytometry detection of the expression levels of monocyte marker Ly6C, M1 marker CD86 and M2 marker CD163 in liver tissues from indicated mice. MFI, mean fluorescence intensity. **p < .01; *p < .05.

Next, we explored the role of ALKBH5 in HSC in the RILF model. We used HBAAV‐GFAP‐shALKBH5 to knock down ALKBH5 in HSC. As expected, HBAAV‐GFAP‐shALKBH5 could effectively down‐regulate the expression of ALKBH5/TIRAP axis in HSC (Figure 4A,B). HBAAV‐GFAP‐shALKBH5 inhibited α‐SMA and collagen 1 expression in liver tissue and HSC, decreased α‐SMA expression around the vessel and reduced perivascular collagen deposition (Figure 4C,D). In addition, HBAAV‐GFAP‐shALKBH5 treatment down‐regulated CCL5 expression in liver tissue and HSC (Figure 4E). As expected, the silence of ALKBH5 suppressed the infiltration of IBA1^+^CLEC4F^−^ monocytes in perivascular tissue and Ly6C^+^ monocytes in liver (Figure 4F,H). Notably, M2 marker CD163 decreased significantly, while M1 marker CD86 decreased slightly after ALKBH5 knockdown (reduced 51.23% vs. 17.95%; Figure 4G,H). In summary, ALKBH5 inhibition in HSC alleviates RILF and suppresses monocyte infiltration and M2 polarisation.

### 2.5 IR‐HSC‐educated monocytes promote HSC activation

**FIGURE 5:**
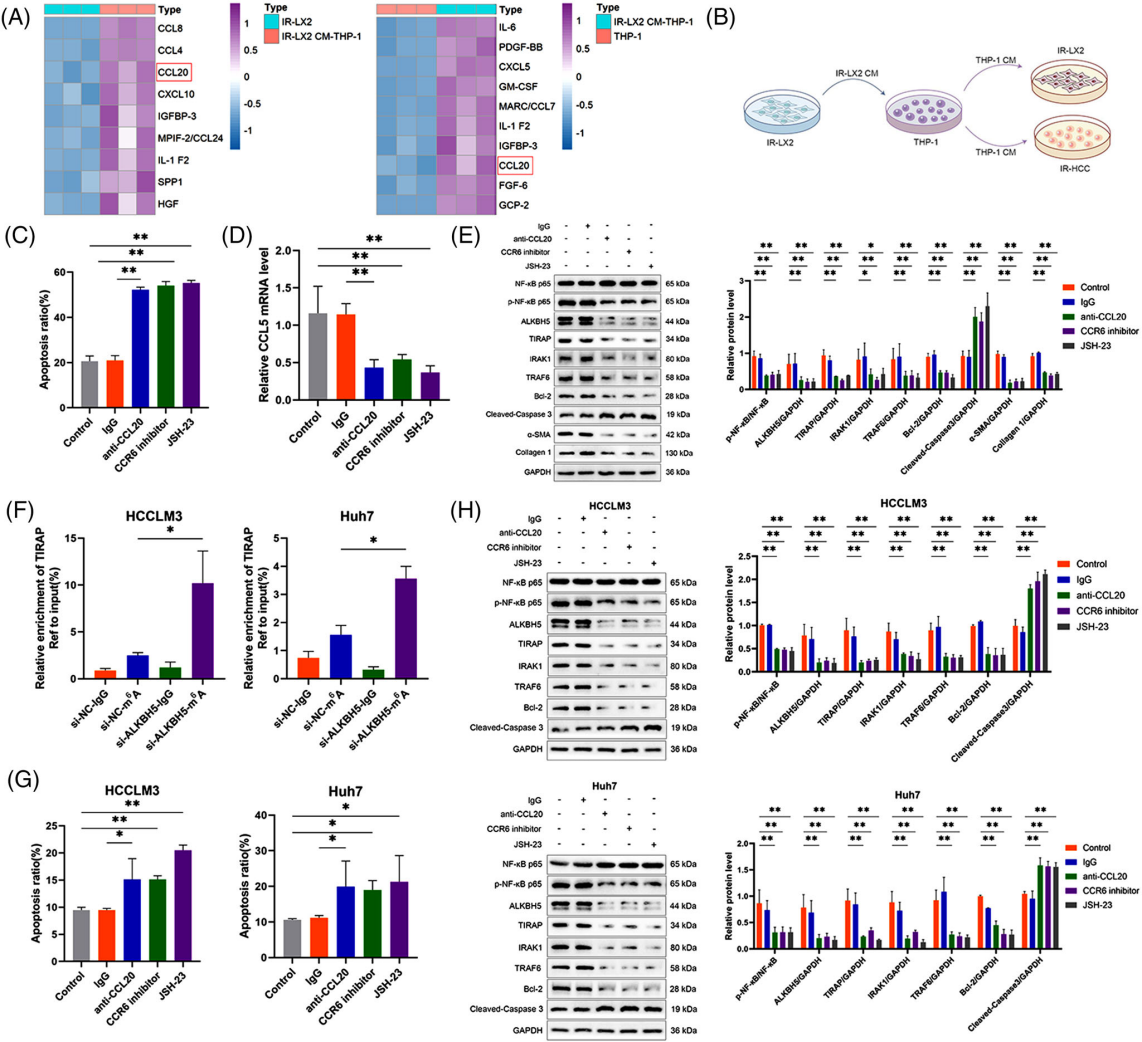
IR‐HSC educates monocyte to promote HSC activation and reduce radiosensitivity of hepatocellular carcinoma (HCC) cells. (A) Cytokine array detection for the CM from IR‐LX2, THP‐1 and IR‐LX2 CM stimulated THP‐1 cells (IR‐LX2 CM‐THP‐1). (B) Schematic overview of the coculture.THP‐1 CM was collected from IR‐LX2 CM stimulated THP‐1 cells and then co‐cultured with IR‐LX2 cells or IR‐HCC cells. (C) Apoptotic cell detection of IR‐LX2 cells after co‐culturing with THP‐1 CM from (B) in the presence of control IgG, anti‐CCL20 neutralising antibody (10 µg/mL), CCR6 inhibitor (20 µM) or JSH‐23 (50 µM). **p < .01. (D) mRNA level of CCL5 in IR‐LX2 cells treated as (C). **p < .01. (E) Western blot analysis of the related proteins in IR‐LX2 cells treated as (C). **p < .01. (F) MeRIP qRT‐PCR analysis of m^6^A modification of TIRAP in control or ALKBH5‐silenced IR‐HCC cells (HCCLM3 and Huh7) using anti‐IgG and anti‐m^6^A antibodies. *p < .05. (G) Apoptotic cell detection of IR‐HCC cells (HCCLM3 and Huh7) after co‐culturing with THP‐1 CM from (B) in the presence of control IgG, anti‐CCL20 neutralising antibody (10 µg/mL), CCR6 inhibitor (20 µM) or JSH‐23 (50 µM). **p < .01; *p < .05. (H) Western blot analysis of the related proteins in IR‐HCC cells (HCCLM3 and Huh7) treated as (G). **p < .01.

Next, we explored the effect of IR‐HSC‐educated monocytes on HSC. We found that the CM from IR‐LX2 CM‐stimulated THP‐1 cells could reduce the cell apoptosis, promote fibrotic markers expression and significantly up‐regulate the expression of ALKBH5, TIRAP, BCL2 and CCL5 of IR‐LX2 cells (Figure S6A–C). ALKBH5 knockdown in IR‐LX2 cells could reverse these effects (Figure S6D–F), indicating that these effects were correlated with the further upregulation of ALKBH5. Subsequently, the medium from IR‐LX2, blank THP‐1 and IR‐LX2 CM‐stimulated THP‐1 (IR‐LX2 CM‐THP‐1) was collected for cytokine microarray detection. Compared with the medium of IR‐LX2 and blank THP‐1, CCL20 was significantly increased in the medium of IR‐LX2 CM‐THP‐1 (Figure 5A). Consistently, CCL20 expression was significantly up‐regulated in THP‐1 cells after co‐culturing with IR‐LX2 CM (Figure S6G1). Additionally, CCL5 increased CCL20 expression in a dose‐dependent manner, while maraviroc blockade could reverse it (Figure S6G2). Co‐culturing with ALKBH5‐silenced IR‐LX2 CM decreased CCL20 expression in THP‐1 cells (Figure S6G3). Moreover, CCL20 expression was decreased in MK2206 pretreated THP‐1 cells co‐cultured with IR‐LX2 CM (Figure S6G3). Similar results were observed in the co‐culture of mouse IR‐HSC CM and BMDMs (Figure S8A–D). These results suggested that CCL5 secreted by IR‐HSC promotes monocyte to produce CCL20 through the AKT pathway.

Moreover, we found that CCL20 promoted NF‐κB activation and up‐regulated ALKBH5 expression in a dose‐dependent manner in IR‐LX2 cells (Figure S7A). CCL20 stimulation reduced the cell apoptosis and promoted CCL5 secretion as well as cell activation of IR‐LX2 (Figure S7B–D). Notably, CCR6 inhibitor down‐regulated the expression of CCL5 and ALKBH5/TIRAP axis and inhibited cell activation and NF‐κB pathway in CCL20‐treated IR‐LX2 cells (Figure S7E,F). In order to determine whether CCL20 regulates ALKBH5 expression through NF‐κB, LX2 cells were treated with CCL20 combined with JSH‐23. The results showed that JSH‐23 inhibited NF‐κB activation and ALKBH5/TIRAP axis in CCL20‐treated IR‐LX2 cells (Figure S7F). In addition, ALKBH5 knockdown inhibited NF‐κB activation and TIRAP downstream effectors expression, which could be partially reversed by CCL20 (Figure S7G). Furthermore, we collected IR‐LX2 CM‐stimulated THP‐1 CM to co‐culture with IR‐LX2 for validation (Figure 5B). The results showed that CCL20 neutralisation antibody, CCR6 inhibitor or JSH‐23 treatment increased cell apoptosis but reduced the expression of CCL5, cell activation markers and ALKBH5/TIRAP axis (Figure 5C–E). Similar results were observed in the mouse IR‐HSC stimulated by CCL20 or co‐cultured with IR‐HSC CM‐stimulated BMDM CM (Figure S8E–I). In summary, blocking the CCL20/CCR6 axis inhibits HSC activation and CCL5 production and down‐regulates the expression of ALKBH5/TIRAP axis mediated by NF‐κB activation in IR‐HSC.

### 2.6 IR‐HSC‐educated monocytes reduce radiosensitivity of HCC cells

Next, we explored the effect of IR‐LX2 CM‐stimulated THP‐1 CM on the radiosensitivity of HCC cells. We found that IR‐LX2 CM‐stimulated THP‐1 CM reduced the apoptosis of irradiated HCC (IR‐HCC) cells and up‐regulated Bcl‐2 expression (Figure S9A,B). Interestingly, ALKBH5 expression increased in IR‐HCC cells after co‐culture (Figure S9B). In IR‐HCC cells, ALKBH5 knockdown promoted cell apoptosis and inhibited cell proliferation (Figure S9C,D), suggesting that ALKBH5 inhibition increases the radiosensitivity of HCC cells. Notably, CCL20 stimulation reduced the apoptosis of IR‐HCC cells (Figure S9E). Similar to the effect of CCL20 on HSC, CCL20 also promoted NF‐κB activation and up‐regulated ALKBH5 expression in a dose‐dependent manner in IR‐HCC cells (Figure S9F). This finding inspired us to speculate whether ALKBH5‐mediated TIRAP m^6^A modification and expression also exist in HCC cells. MeRIP confirmed that ALKBH5 knockdown promoted m^6^A modification of TIRAP in HCC cells (Figure 5F). CCR6 inhibitor or JSH‐23 inhibited NF‐κB activation and ALKBH5/TIRAP axis in CCL20‐stimulated HCC cells (Figure S9G,H). CCL20 stimulation could partially reverse the effect that ALKBH5 knockdown inhibited the expression of TIRAP and downstream effectors in IR‐HCC cells (Figure S9G,H). Furthermore, in the co‐culture of IR‐LX2 CM‐stimulated THP‐1 CM and IR‐HCC cells, CCL20 neutralising antibody, CCR6 inhibitor or JSH‐23 treatment increased cell apoptosis and inhibited ALKBH5/TIRAP axis (Figure 5G,H). These results suggested that blocking the CCL20/CCR6 axis increases HCC radiosensitivity by inhibiting NF‐κB /ALKBH5/TIRAP axis.

Moreover, we also eliminated the effect of CCL5 expression of HCC cells on monocyte migration and polarisation. Irradiation did not affect CCL5 expression in HCC cells (Figure S10A). IR‐HCC cells secreted a small amount of CCL5, and CCL20 stimulation could not induce HCC cells to produce CCL5 (Figure S10B). In the co‐culture of IR‐LX2 CM‐stimulated THP‐1 CM and IR‐HCC cells, CCL20 neutralising antibody, CCR6 inhibitor or JSH‐23 treatment had no significant effect on CCL5 production (Figure S10C). In addition, the silence of ALKBH5 in IR‐HCC cells did not affect CCL5 expression (Figure S10D). Furthermore, the CM from CCL5‐silenced IR‐HCC cells had no effect on the migration and polarisation of THP‐1 cells (Figure S10E,F). These results suggested that CCL5 expression of HCC cells has no effect on monocyte migration and polarisation.

### 2.7 Blocking ALKBH5/CCR6 axis improves HCC radiosensitivity in mice

**FIGURE 6:**
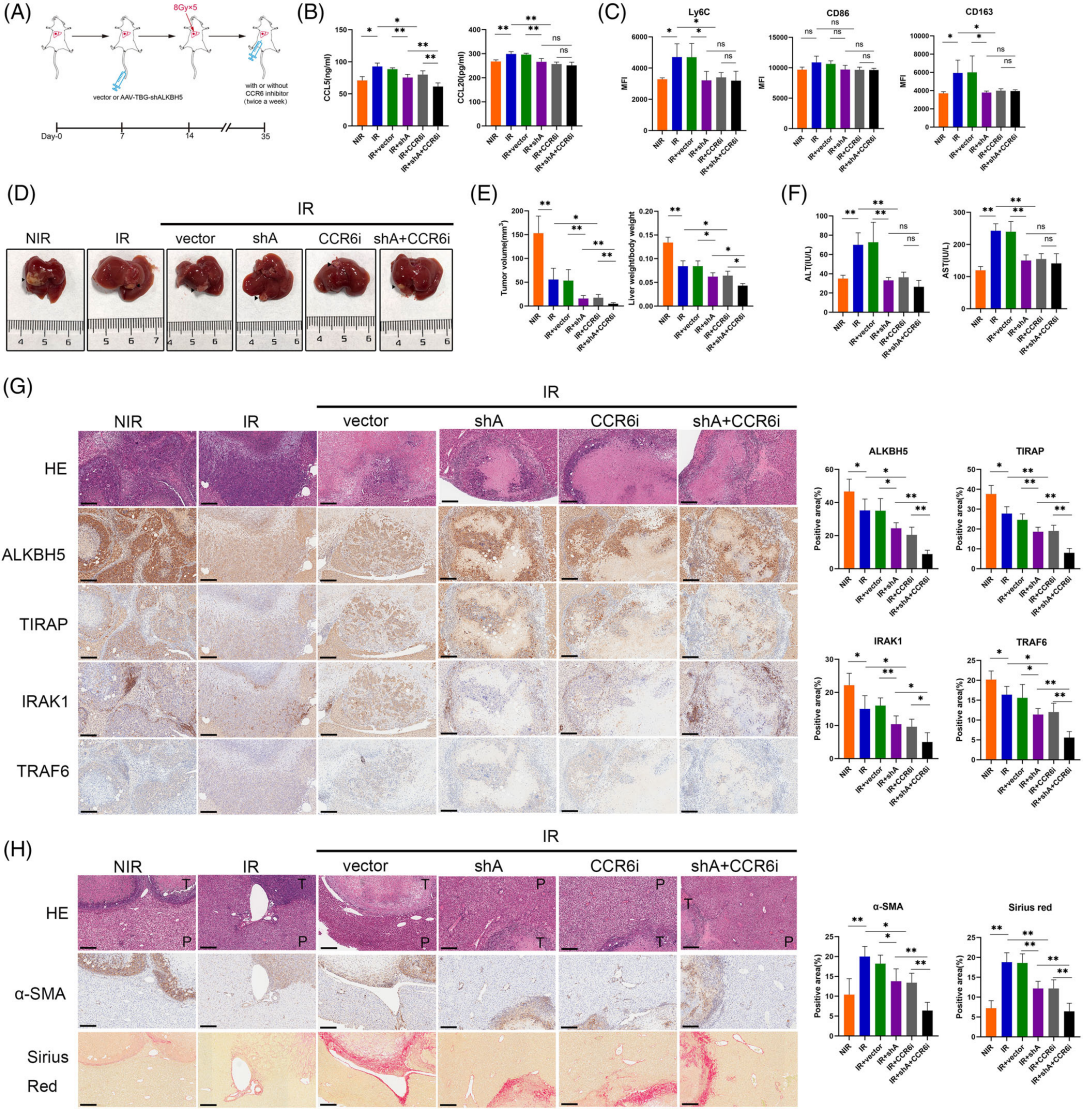
Blocking ALKBH5‐CCR6 axis improves radiosensitivity of HCC and alleviates RILF in mice. (A) Schematic overview of the treatment of the HCC orthotopic xenografts in mice. (B) Enzyme linked immunosorbent assay (ELISA) detection of CCL5 and CCL20 in mice serum after the indicated treatments. **p < .01; *p < .05. NIR, non‐irradiation; IR, irradiation; shA, HBAAV‐TBG‐shALKBH5; CCR6i, CCR6 inhibitor. (C) Flow cytometry detection of the expression levels of monocyte marker Ly6C, M1 marker CD86 and M2 marker CD163 in liver tissues from indicated mice. MFI, mean fluorescence intensity. *p < .05. (D) Representative images of orthotopic tumours in mice liver after the indicated treatments. (E) The tumour volume (left panel) and the ratio of liver weight to body weight (right panel) in mice after the indicated treatments. **p < .01; *p < .05. (F) The serum levels of ALT and AST in mice after the indicated treatments. **p < .01. (G) Hematoxylin eosin (HE) and IHC staining of ALKBH5 and downstream proteins in orthotopic tumours of mice. **p < .01; *p < .05. Scale bar: 200 µm. (H) HE, IHC staining of α‐SMA and Sirius red staining in orthotopic tumour and paratumour liver tissue of mice. **p < .01; *p < .05. Scale bar: 200 µm. T, tumour; P, paratumour.

HBAAV‐TBG‐shALKBH5 or vector was injected through the tail vein 1 week before irradiation, CCR6 inhibitor was injected intraperitoneally twice a week after irradiation, and the experiment was completed 3 weeks after the last irradiation (Figure 6A). Treatment of HBAAV‐TBG‐shALKBH5 or/and CCR6 inhibitor significantly reduced the serum level of CCL5 and CCL20, suppressed the infiltration of Ly6C^+^ monocytes and M2 macrophage but not M1 macrophage in liver tissue (Figure 6B,C). Additionally, treatment of HBAAV‐TBG‐shALKBH5 or/and CCR6 inhibitor markedly decreased tumour volume and liver/body weight ratio, promoted tumour necrosis and inhibited ALKBH5/TIRAP axis in tumour (Figure 6D,E,G). Moreover, the serum level of alanine aminotransferase (ALT) and aspartate aminotransferase (AST) increased markedly in irradiated mice but was significantly attenuated by HBAAV‐TBG‐shALKBH5 or/and CCR6 inhibitor treatment (Figure 6F). After combined treatment, α‐SMA and collagen deposition around the vessel and tumour decreased (Figure 6H). In summary, ALKBH5 inhibition can reduce monocyte infiltration and M2 polarisation, increase HCC radiosensitivity and alleviate RILF.

### 2.8 High expression of ALKBH5 and TIRAP are associated with RILI and poor radiotherapy response of HCC

**FIGURE 7:**
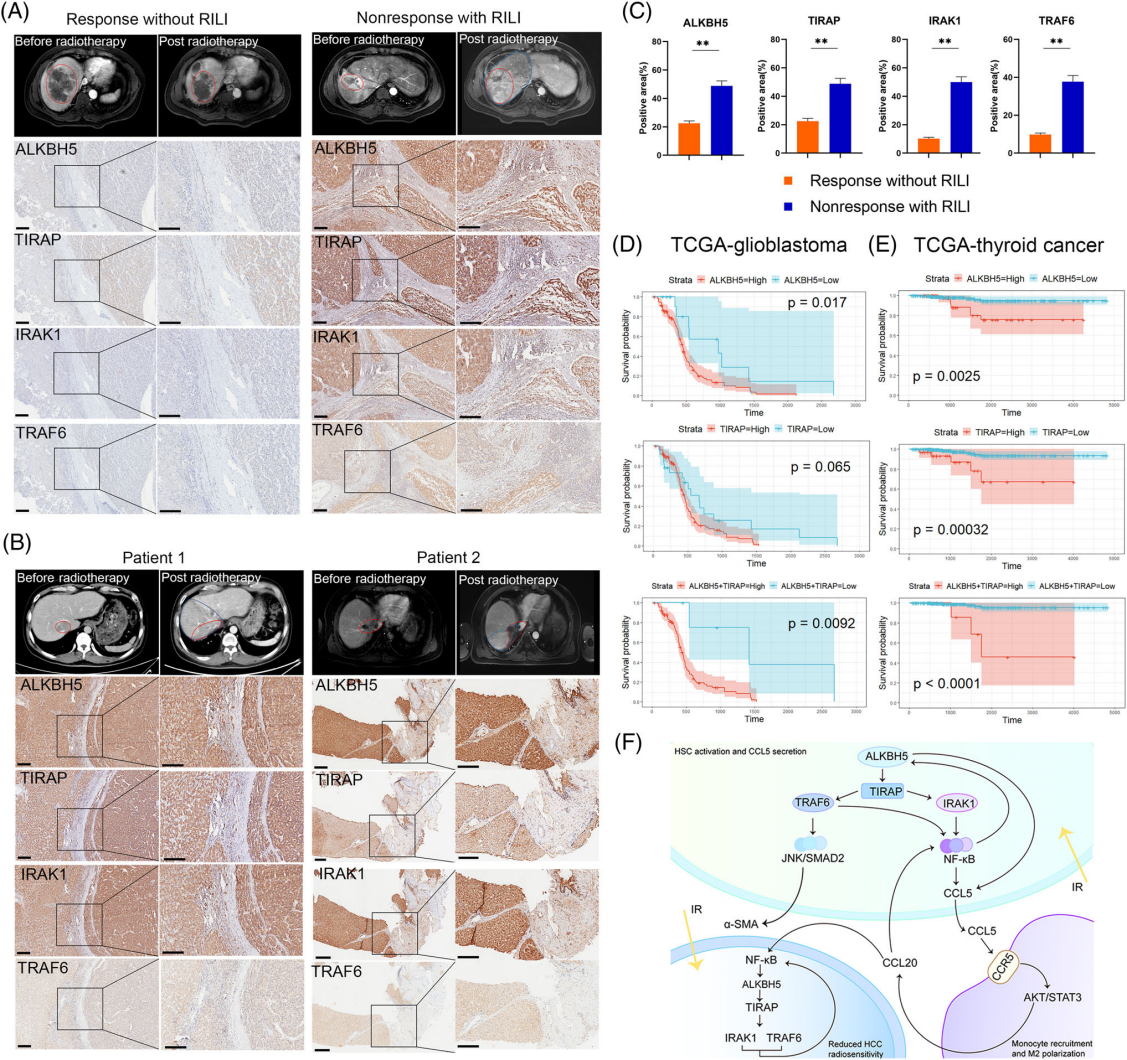
High expression of ALKBH5 and TIRAP correlate with radiation‐induced liver injury (RILI) and poor radiotherapy response of HCC. (A) Representative magnetic resonance imaging (MRI) images before and post radiotherapy were collected in HCC patients with radiotherapy response without RILI or radiotherapy nonresponse with RILI. The red line indicated the tumour and the blue line indicated the RILI area. IHC staining of tumour and paratumour liver tissue from corresponding patients who received surgical resection before radiotherapy were displayed. (B) Representative computed tomography (CT) or MRI images before and post radiotherapy were collected in two HCC patients with radiotherapy nonresponse and RILI. The red line indicated the tumour and the blue line indicated the RILI area. IHC staining of tumour and paratumour liver tissue from corresponding patients who received surgical resection or liver biopsy post radiotherapy was displayed. (C) Statistics of indicated targets between radiotherapy response without RILI and nonresponse with RILI in HCC patients as indicated in (A, B). **p < .01. (D‐E) The prognostic value of ALKBH5 and TIRAP for glioblastoma (D) and thyroid cancer (E) patients receiving radiotherapy from TCGA database. (F) A schematic model of the mechanism by which irradiation up‐regulates ALKBH5 in HSC to mediate monocyte recruitment and polarisation and form positive feedback to promote radiation induce liver fibrosis and reduce radiosensitivity of HCC.

In order to better understand the relationship between ALKBH5/TIRAP axis and RILI and HCC radiosensitivity, we collected samples from patients responding to radiotherapy without concomitant RILI and patients showing nonresponse to radiotherapy but with concomitant RILI. High expression of ALKBH5/TIRAP axis was detected in tumour and paratumour tissues before or post radiotherapy from HCC patients who showed non‐response to radiotherapy but with concomitant RILI, compared to those patients responding to radiotherapy without RILI (Figure 7A–C), suggesting that these indicators may be used as potential markers for predicting radiotherapy response and complications of HCC. Moreover, we found that high expression of ALKBH5 and/or TIRAP was significantly associated with the poor overall survival of glioblastoma and thyroid cancer patients receiving radiotherapy from TCGA database(Figure 7D–E), suggesting ALKBH5 and TIRAP have prognostic value for certain patients receiving radiotherapy.

## 3 DISCUSSION

Studies on radiotherapy‐induced m^6^A modification, especially ALKBH5‐mediated demethylation modification, are still rarely reported. Radiation‐induced ALKBH5 mediated m^6^A demethylation of IL‐6 mRNA and reduced IL‐6 production to alleviate radiation pneumonitis.^26^ In this study, radiation‐induced ALKBH5 expression in HSC mediated m^6^A demethylation of TIRAP mRNA and enhanced its expression, thereby promoting the activation of downstream NF‐κB and JNK/Smad2 pathways, leading to HSC activation and promoting the occurrence of RILF. Our study revealed for the first time the role of ALKBH5‐mediated m^6^A modification in radiation‐induced HSC activation. However, ALKBH5 responded differently to irradiated and non‐irradiated conditions. ALKBH5 is down‐regulated in liver tissues of a CCL4‐induced mouse liver fibrosis model and in TGF‐β‐stimulated activated HSC in vitro, whereas high expression of ALKBH5 ameliorates liver fibrosis and inhibits HSC activation.^27^ The distinct outcomes are largely attributable to differences in experimental intervention conditions, resulting in different expression status of ALKBH5. Recent findings demonstrate that hypoxia as well as some epigenetic regulators, transcription factors and noncoding RNAs are involved in regulating the abnormal expression of ALKBH5.^28^, ^29^, ^30^, ^31^ These regulators constitute a complex upstream regulatory network for ALKBH5. Under different intervention conditions, these regulatory factors may acquire different regulatory advantages, resulting in different expression status of ALKBH5 in different disease backgrounds. ALKBH5 mediates the demethylation of target genes, and the expression of target genes is controlled by some regulatory factors, such as m^6^A recognition proteins YTHDF1/2/3 and IGF2BP1 as well as HuR.^23^ Therefore, in different disease backgrounds, ALKBH5 high expression may lead to different target gene expression profiles, thereby affecting the downstream regulatory effects of ALKBH5. This also provides a certain explanation for the different regulatory effects of ALKBH5 on HSC activation between this study and the previous report.

Our further study revealed that ALKBH5 regulates the CCL5 production from IR‐HSC through multiple pathways to promote monocyte infiltration and M2 polarisation, and the educated monocytes can in turn promote HSC activation and CCL5 production. A positive feedback regulatory loop exists between IR‐HSC and monocyte, which provides favorable conditions for IR‐HSC to maintain an activated phenotype. These findings provide a partial explanation for the long‐term persistence of RILF in both clinical patients and mouse models, suggesting that blocking the ALKBH5/CCL5 axis may be a potential strategy to prevent RILF.

Another interesting finding was that IR‐HSC‐educated monocyte can also promote ALKBH5 expression and suppress radiosensitivity of IR‐HCC cells. At present, the relationship between ALKBH5 and radiosensitivity of tumours has not been reported. Our study confirmed that ALKBH5 knockdown increases the radiosensitivity of HCC. Except for ALKBH5, related studies have reported the role of some m^6^A regulators, such as METTL3 and FTO, in the induction of tumour radiation resistance.^32^, ^33^ However, there is still partial disagreement regarding ALKBH5 and the response to HCC treatment. A previous study showed that ALKBH5 is down‐regulated in HCC and exerts an inhibitory effect on HCC proliferation.^34^ Another study showed that ALKBH5 is up‐regulated in hepatitis B virus (HBV) HCC tissues and has a tumour‐promoting effect and predicts a poor prognosis.^35^ This is similar to the different regulatory roles of ALKBH5 on HSC activation that we discussed earlier. Many tumours, including HCC, are complex and heterogeneous diseases.^36^ Independent studies of different tumour subtypes or the use of different incidence or intervention models often draw very different conclusions. Considering that ALKBH5 regulates a large number of target genes with different functions, it is not surprising that the different behaviours of ALKBH5 in different cellular contexts are related to its specific regulatory network, thus resulting in different regulatory effects.^23^ Notably, mRNA or protein expression of ALKBH5 is not synonymous with m^6^A demethylase activity. Because ALKBH5 is a member of Fe(II)/2‐oxoglutarate‐dependent dioxygenases AlkB protein family, oxygen, ferrous iron and 2‐oxoglutarate may affect the enzyme activity of ALKBH5.^17^, ^23^ The prediction of RILI and radiosensitivity of HCC based on ALKBH5 expression alone may not be reliable. Therefore, in addition to ALKBH5, we should also pay attention to the relationship between the overall functional activities of target genes modified by ALKBH5 and the development of disease. In this study, we found that ALKBH5, TIRAP and its downstream IRAK1 and TRAF6 were highly expressed in liver tissues of HCC patients with poor response to tumour radiotherapy and concomitant RILI, and functional experiments confirmed that the activation of TIRAP/NF‐κB pathway mediated by ALKBH5 significantly promoted RILF and decreased the radiosensitivity of HCC. It indicated that the abnormal activation of ALKBH5/TIRAP regulatory axis in the irradiation background is an adverse factor in predicting the treatment efficacy of HCC. This may also partly explain why 14.7% of patients are still complicated with RILI after using advanced stereotactic radiotherapy technology. In addition, the TCGA database showed that co‐high expression of ALKBH5 and TIRAP is associated with poor prognosis in patients with glioblastoma and thyroid cancer receiving radiotherapy. It also enlightens us that in the future, we can consider building a prediction model of tumour radiotherapy response and RILI based on ALKBH5/TIRAP regulatory axis genes and designing relevant targeted therapy strategies rather than simply based on ALKBH5 expression.

In conclusion, this study revealed that radiation‐induced upregulation of ALKBH5 in HSC mediates TIRAP/NF‐κB pathway activation to promote HSC activation, while ALKBH5 regulates CCL5 secretion to promote monocyte recruitment and M2 polarisation, which further promotes ALKBH5 expression and activation of TIRAP/NF‐κB pathway in HSCs and HCC cells, which in turn aggravates RILF and reduces HCC radiosensitivity (Figure 7F). In addition, polarised monocytes promote HSC to generate more CCL5, resulting in positive feedback regulation. This study proposes that ALKBH5 can not only serve as a microenvironmental regulator but also as a radiosensitisation target, which is the key innovation of this study.

## 4 MATERIALS AND METHODS

### 4.1 In vivo animal studies

Male C57BL/6 mice (6‐7 weeks old and 16–20 g) were maintained under pathogen‐free conditions in the Experimental Animal Center of Southern Medical University. All animal experiments were specifically approved by Southern Medical University Experimental Animal Ethics Committee. ALKBH5‐short hairpin RNA (shRNA) encapsulated by adeno‐associated virus containing HSC‐specific promoter of glial fibrillary acidic protein (HBAAV‐GFAP‐shALKBH5, 1×10^12^ vg/mL) or liver‐specific promoter (HBAAV‐TBG‐shALKBH5, 1×10^12^ vg/mL) and vector (1×10^12^ vg/mL) were designed and synthesised by Hanbio Biotechnology. For mice irradiation, the right liver lobe of mice was irradiated with X‐rays (8 Gy × 5 times, once a day). For the evaluation of maraviroc in modulating monocyte infiltration, intraperitoneal injection of maraviroc (10 mg/kg) was performed once a day after the initiation of irradiation. For the evaluation of ALKBH5 expression of HSC in modulating RILF and monocyte infiltration, HBAAV‐GFAP‐shALKBH5 or vector (100 uL per mouse) was injected once a week before irradiation. Three weeks after the last irradiation, the mice were sacrificed, and liver tissues were collected for histological, immunohistochemistry and immunofluorescence analysis and flow cytometry. The remaining fresh liver tissues were used for qRT‐PCR and western blot analysis.

Orthotopic HCC models were established according to the method described previously.^37^ Hepa 1–6 cells (5×10^5^) were suspended in 20 µL PBS and injected into the right liver lobe of C57BL/6 mice. After 2 weeks, the mice were randomly grouped and the right liver lobe was irradiated with X‐rays (8 Gy×5 fractions, once a day), and a non‐irradiated group was set up as a control. The irradiation groups were treated as follows: (1) single irradiation; (2) tail vein injection of HBAAV‐TBG‐shALKBH5 (100 uL per mouse) once a week before irradiation; (3) tail vein injection of vector (100 uL per mouse) once a week before irradiation; (4) intraperitoneal injection of CCR6 inhibitor (10 mg/kg, MedChemExpress) twice a week after initiation of irradiation; (5) tail vein injection of HBAAV‐TBG‐shALKBH5 combined with an intraperitoneal injection of CCR6 inhibitor. Tumour volume and liver/body weight ratio were evaluated at 3 weeks after the last irradiation. Tumour volume was calculated using the formula: *tumour* *volume* (mm^3^) = (smallest diameter^2^ × largest diameter)/2.^38^ The venous blood of mice was drawn from the eye orbit of mice, and then the mice were sacrificed at 3 weeks after the last irradiation. The levels of AST and ALT in serum were detected by an automatic biochemistry analyzer. The right liver lobes were collected for histological, immunohistochemistry and immunofluorescence analysis and for monocyte–macrophage detection by flow cytometry.

### 4.2 Patient samples

Seventeen HCC samples were collected from patients who received radiotherapy for intrahepatic tumour during 2018–2021 (for detailed patient information, see Table S1). Among them, 15 samples were collected before radiotherapy, and two samples were collected post radiotherapy. The radiotherapy doses ranged from 40 to 60 Gy with 5–20 fractions. All the patients conducted the computed tomography (CT) or magnetic resonance imaging (MRI) image examination before and after radiotherapy. The treatment effects were evaluated within 6 months after the last irradiation. Informed consent in writing was obtained from each patient. The study protocol was approved by the review committee of the Nanfang Hospital of Southern Medical University.

The treatment response was assessed according to the Modified Response Evaluation Criteria in Solid Tumour. After radiotherapy, it was defined as ‘response’ that the tumour volume was significantly reduced, the signs of ‘fast in and fast out’ were lost or the tumour had no obvious enhancement in the arterial phase, while the increased tumour volume, local recurrence or new lesions were defined as ‘nonresponse’. For image evaluation of RILI, in the area of focal oedema or congestion caused by veno‐occlusive disease and hepatic fibrosis after irradiation, the irradiated area usually displays hypoattenuation on unenhanced CT. On contrast‐enhanced CT, it could show hyperdensity in all enhanced phases, hypodensity in the arterial and portal venous phases or isodensity in all enhanced phases. For MRI, the irradiated area usually exhibits hypointensity on T1‐weighted images, hyperintensity on T2‐weighted images, and a defined hypointense area on the hepato‐biliary phases of gadolinium‐ethoxybenzyl‐diethy‐lenetriamine pentaacetic acid (Gd‐EOB‐DTPA)‐enhanced MRI.^10^

The rest of the materials and methods are included in the Supplementary Information.

## CONFLICT OF INTEREST STATEMENT

The authors declare no competing financial interests.

## Supporting information

Supplementary Information
