# Supplementary material for: ALKBH5‐mediated m6A demethylation of TIRAP mRNA promotes radiation‐induced liver fibrosis and decreases radiosensitivity of hepatocellular carcinoma
Source: Clin Transl Med. 2023 Feb 15;13(2):e1198. doi: 10.1002/ctm2.1198 (PMC9931500; doi:10.1002/ctm2.1198)
Supplement: Supplementary file 1 — Supplementary Information [file CTM2-13-e1198-s001.docx]

**Supplementary Information**

**ALKBH5-mediated m^6^A demethylation of TIRAP mRNA promotes radiation-induced liver fibrosis and decreases radiosensitivity of hepatocellular carcinoma**

Yuhan Chen^1^, Peitao Zhou^1^, Yixun Deng^2^, Xinni Cai^2^, Mingrui, Sun^2^, Yining Sun^1^, Dehua Wu^1^

^1^Department of Radiation Oncology, Nanfang Hospital, Southern Medical University, Guangzhou, Guangdong Province, 510515, China

^2^The First School of Clinical Medicine, Southern Medical University, Guangzhou, Guangodng Province, 510515, China

**Supplementary Methods**

**Cell culture, transfection and irradiation**

Human HSC cell line LX2 was purchased from Jennio Biotech (Guangzhou, China). Human HCC cell lines Huh7 and HCCLM3 were obtained from Shanghai Advanced Research Institute, Chinese Academy of Sciences. Cells were cultured in Dulbecco’s Modified Eagle’s Medium (DMEM) (pH 7.4) supplemented with 10% (v/v) fetal bovine serum (Cat no. FSP500, Excell Bio, Suzhou, China). Small interfering RNAs (siRNAs) for ALKBH5, TIRAP, YTHDF2 and CCL5 were purchased from RiboBio Biotech (Guangzhou, China). The TIRAP-overexpressing plasmid was purchased from Genechem (Shanghai, China). 50 nM siRNA or 1 ug plasmid was transfected into cells in 24-well plates by using Lipofectamine 3000 reagent (Cat no. L3000015, Invitrogen, California, USA) according to the manufacturer's instruction. The stable expression of ALKBH5 in LX2 cells was infected with Lentivirus-ALKBH5 (WZ Biosciences, Jinan, China). For irradiation, cells were treated with the indicated dose of X-ray using a linear accelerator (Varian Clinac 23EX Linear Accelerator, California, USA). The machine parameters for irraidation were photon beam energy of 6 MV, dose rate of 3 Gy/min, source surface distance of 100 cm and the gantry at 180°.

**Isolation of primary HSCs and bone marrow derived monocytes (BMDMs)**

The isolation of primary HSCs was performed according to previously described methods[1]. HSCs were isolated from the indicated C57BL/6 mice by in situ perfusion with Hank’s solution containing 0.05% collagenase IV (Cat no. V900893, Sigma, Michigan, USA) and 0.01% DNase I (Cat no. D5025, Sigma). After digestion and filtration, the acquired cell suspension was centrifuged at 400 rpm for 5 min at 4 °C to remove the pellet containing primary hepatocytes. The supernatants were centrifuged at 2000 rpm for 10 min at 4 °C to collect non-parenchymal liver cells. And the primary HSCs were harvested through density gradient centrifugation. Isolated HSCs were cultured in DMEM (pH 7.4) supplemented with 10 μg/ml streptomycin sulfate, 100 μg/ml penicillin G, and 10% (v/v) fetal bovine serum.

For the isolation of BMDMs, the femur and tibia of C57BL/6 mice were rinsed with PBS, and the bone marrow cell suspension was collected. The purified BM mononuclear cells were obtained through standard Ficoll-Paque density gradient centrifugation. After that, the cells were treated with 20 ng/ml M-CSF (Cat no. 315-02, PeproTech, New Jersey, USA) at 37°C in a humidified atmosphere of 5% CO_2_ for 5 days. Those unadherent cells suspended in cell culture medium were collected as monocytes[2].

**Cell proliferation and apoptosis**

Cell viability was detected by using the Cell Counting Kit-8 (CCK-8) (Dojindo, Kumamoto, Japan) according to the manufacturer’s protocol. The optical density was recorded at 450 nm through a microplate reader. Cell apoptosis was determined by flow cytometry . According to the manufacturer’s protocol, cells were harvested at the indicated time and resuspended in the binding buffer and stained with the PI and Annexin V-AF647 using Annexin V-AF647/PI Apoptosis Kit (Cat no. 100-102, Goonie, Guangzhou, China).

**Quantitative reverse transcription polymerase chain reaction (qRT-PCR)**

After extraction of total RNA using TRIzol (Cat no. 15596026, Invitrogen), cDNA was synthesized using HiScript® III RT SuperMix for qPCR (+gDNA wiper) (Cat no. R323-01, Vazyme, Nanjing, China), and then Taq Pro Universal SYBR qPCR Master Mix (Cat no. Q712-02, Vazyme) was used in real-time detection system for quantitative RT-PCR detection. The primer sequences are shown in *Table S2*. GAPDH was used as the internal control for the detection of mRNA, and the relative expression level was calculated by using 2^-△△Ct^ method[3].

**Western blot**

Protein was isolated using RIPA lysis buffer (Cat no. P0013B, Beyotime, Shanghai, China) containing phenylmethylsulfonyl fluoride (PMSF) (Cat no. ST507, Beyotime). The protein extracts were transferred to a 10%-15% sodium dodecyl sulfate-polyacrylamide gel for electrophoretic separation, and then the proteins on the gel were transferred to PVDF membranes (Millipore). After blocking with 5% nonfat milk for 1 h, the membranes were incubated with the corresponding primary antibody overnight at 4°C. Antibodies used in this study were listed in *Table S3*. The membranes were then incubated with horseradish peroxidase-conjugated secondary antibody (Cat no. FDR007 & Cat no. FDM007, Fudebio, Hangzhou, China) for 1 h at room temperature. Protein bands were visualized using a chemiluminescence system (BLT GelView 6000 Pro, Boluteng Bio, Guangzhou, China). GAPDH was served as the internal control.

**Methylated RNA immunoprecipitation sequencing (MeRIP-seq)**

After extraction of total RNA, the quality and quantity of RNA were analyzed using a Qubit RNA HS assay kit (Cat no. Q32852, Invitrogen). Then the RNA was fragmented into ~100nt long oligonucleotides using ZnCl_2_. The distribution of fragmented size was then determined on the Agilent 2100 Bioanalyzer using the Agilent RNA 6000 kit. Magna ChIP protein A/G magnetic beads (Cat no. 88802, Thermo Fisher Scientific, [Massachusetts](https://www.bing.com/ck/a?!&&p=0ea10e60cd2812deJmltdHM9MTY2MjQyMjQwMCZpZ3VpZD0xMzZjMmIxOS03ZDE4LTY1ZTUtMDlmMi0zYjNlN2MzNjY0MjQmaW5zaWQ9NTQyNA&ptn=3&hsh=3&fclid=136c2b19-7d18-65e5-09f2-3b3e7c366424&u=a1L3RyYXZlbC9wbGFjZS1pbmZvcm1hdGlvbj9xPU1hc3NhY2h1c2V0dHMmU0lEPTg0NTIxOWQ1LTM2NTAtNDE5OS1iOTI2LTk2NGNhMjdjODYzYyZmb3JtPURFU1RNTA&ntb=1), USA) were incubated with m^6^A antibodies (Cat no. ABE572, Sigma) and then the mixture was incubated with the MeRIP reaction mixture containing the fragmented RNA. Next, the eluted and purified RNA was used for RNA-seq library construction by using a NEBNext Ultra RNA library prep kit (Cat no. E6177, NEB, [Massachusetts](https://www.bing.com/ck/a?!&&p=0ea10e60cd2812deJmltdHM9MTY2MjQyMjQwMCZpZ3VpZD0xMzZjMmIxOS03ZDE4LTY1ZTUtMDlmMi0zYjNlN2MzNjY0MjQmaW5zaWQ9NTQyNA&ptn=3&hsh=3&fclid=136c2b19-7d18-65e5-09f2-3b3e7c366424&u=a1L3RyYXZlbC9wbGFjZS1pbmZvcm1hdGlvbj9xPU1hc3NhY2h1c2V0dHMmU0lEPTg0NTIxOWQ1LTM2NTAtNDE5OS1iOTI2LTk2NGNhMjdjODYzYyZmb3JtPURFU1RNTA&ntb=1), USA). Finally, the input samples and the m^6^A IP samples were analyzed by deep sequencing on the Illumina Novase 6000 platform.

**MeRIP-qRT-PCR**

The m^6^A modification on individual transcripts was analyzed by MeRIP assay through the Magna MeRIP m^6^A Assay Kit (Cat no. 17-10499, Millipore, Darmstadt, Germany). In brief, the RNA was chemically fragmented into 100 nucleotides or smaller fragments. The magnetic beads were pretreated with 10 μg anti- m^6^A antibody (Millipore) or anti-mouse IgG (Millipore) at 37℃ for 30 min. After preparing the MeRIP reaction mixture by using 300 ug of total RNA, the mixture was incubated with the beads-antibody with rotation for 2 h at 4℃. Then the elution buffer containing 20 mM m^6^A was added into the beads to elute the m^6^A-modified RNA fragments. The eluted RNA was purified and then analyzed by qRT-PCR. The percent of input for each MeRIP sample was calculated as %input=2^(CtIP-(Ctinput-log2[10]))^[4].

**Luciferase assay**

LX2 cells were transfected with si-ALKBH5, Lv-ALKBH5 or corresponding negative control. The wild type or the mutated 3’ untranslated region (3’UTR) containing m^6^A binding site of TIRAP, was respectively synthesized and inserted into GV306 vector (Genechem). The above vectors were transfected into cells using Lipofectamine 3000. The firefly and renilla luciferase activities were detected by using the dual-luciferase reporter assay system (Cat no. E1910, Promega, Massachusetts, USA). And the Renilla luciferase functioned as the internal control for luciferase activity. For the ALKBH5 reporter assay, similar steps were performed in the LX2 cells treated or untreated with 50 uM JSH-23 (Cat no. J863496, Macklin, Shanghai, China) and the GV306 vector containing wild-type or the mutated NF-κB p65 binding site in ALKBH5 promoter was used.

**RNA Stability**

LX2 cells transfected with si-ALKBH5, Lv-ALKBH5 or corresponding negative control were treated with 2 mg/ml Actinomycin D (Cat no. A4262, Bo'ao Tuoda, Beijing, China) for 0, 2, 4, 8, 16 or 24 h. Total RNAs were extracted for qRT-PCR analysis.

**Chromatin immunoprecipitation (ChIP) assay**

ChIP assays were conducted according to the instruction of ChIP Assay Kit (Cat no. P2078, Beyotime, Shanghai, China). LX2 cells were treated with JSH-23 (50 uM) for 48 h and then cross-linked using 1% formaldehyde for 10 min at 37℃. After centrifugation, the pellets were subjected to SDS lysis buffer for 10 min and then sonicated. After DNA immunoprecipitation and cross-linked DNA reversal, the purified DNA was detected by qRT‐PCR. The normal rabbit IgG served as the negative control.

**Co-culture of HSCs/HCC cells and monocytes**

Cell migration experiments were performed in 24-well transwell chambers (8 µm pore size, Cat no. 353097, Corning Costar, Maine, USA). Human monocytes THP-1 cells (2 x 10^5^) were added to the upper compartment containing serum-free RPMI1640 medium. Non-irradiated or irradiated LX2 (IR-LX2) cells or IR-LX2 cells with altered ALKBH5 expression levels were cultured with serum-free medium for 72 h, and the above culture medium (CM) was collected for ELISA detection and further experiments. The CM from IR-LX2 (IR-LX2 CM) and control medium (fresh serum-free medium) with or without CCL5 (20 ng/ml, Cat no. RP00321, ABclonal, Wuhan, China) and IR-LX2 CM with CCL5 neutralizing antibody (10 μg/ml, Cat no. 12000-1-AP, Proteintech, Rosemont, USA) were added to the corresponding lower compartment for incubation at 37°C, 5% CO_2_ for 24 h. And the number of cells attached to the lower side of the wells was counted.

For the CCR5 inhibitor maraviroc (Cat no. S2003, MCE, New Jersey, USA) group, after culturing THP-1 cells with maraviroc (5 uM) in culture dishes for 24 h, these cells were transferred to the upper compartment and IR-LX2 CM was added to lower compartment to co-culture for 24 h.

In the differentiation and polarization experiments, THP-1 cells were treated with or without MK2206 (500 nM, Cat no. A3010, APExBIO, Houston, USA) for 24 h. IR-LX2 CM co-cultured with THP-1 cells for 72 h in culture dishes. Then THP-1 cells were collected for further analysis and the culture medium was collected as THP-1 CM. A similar approach was applied to co-culture analysis of primary HSCs and BMDMs. And IR-HSC CM and BMDM CM were obtained in the similar approach as IR-LX2 CM and THP-1 CM.

For the feedback regulatory effects of co-culture conditioned medium, THP-1 CM or control medium (fresh serum-free DMEM medium) with or without CCL20 (200 ng/ml, Cat no. 10485-H07E, ABclonal) and THP-1 CM with CCL20 neutralizing antibody (10 μg/ml, Cat no. ab9829, abcam), CCR6 inhibitor (20 uM, Cat no. HY-112701, MCE) or JSH-23 (50 uM) was added to the culture dish of LX2 or HCC cells (HCCLM3, Huh7) after irradiation by 8 Gy X-ray. After co-culturing for 72 h, LX2 or HCC cells were collected for further analysis and the supernatant was collected for ELISA detection.

**Cytokine array assess**

The cytokine profile in medium was analyzed using Human Cytokine Array G5AAH-CYT-G5 (RayBiotech, Guangzhou, China). Briefly, after blocking slide, 100 μl of each sample was added to each sub-array and then incubated in the incubation chamber with Adhesive film for 2 h at room temperature. After washing, 70 μl of 1X Biotin-conjugated Anti-Cytokines was added to each sub-array and then incubated for 2 h at room temperature. After washing, 70 μl of 1X Streptavidin-Fluor was added to each sub-array and then incubated for 2 h at room temperature. Then, scan the glass chip with Innoscan 300 microarray scanner (Inopsys, Carbonne, France) using cy3 or “green” channel (excitation frequency = 532 nm). Finally, the signal intensity data were analyzed by the RayBio® Analysis Tool software (RayBiotech).

**Enzyme-linked immunosorbent assay (ELISA)**

The concentrations of CCL5 and CCL20 in culture supernatants or serum were detected by ELISA kits (Meimian Industrial, Jiangsu, China) according to the manufacturer’s directions. Absorbance was recorded at 450 nm using a microplate reader.

**Immunohistochemistry (IHC)**

IHC staining was conducted as previously reported[5]. Briefly, tissue sections were deparaffinized, followed by antigen retrieval, and incubated with indicated primary antibodies at their optimal concentration overnight and then incubated with biotin-labeled second antibody and peroxidase-labeled streptavidin. The primary antibodies employed were listed in *Table S3*. Hematoxylin was used for counterstaining. The reaction products were then visualized using a diamidobenzidine tetrahydrochloride (DAB) detection kit (Cell Signaling Technology, CA, USA). ImageScope was used for images capturing (Leica Biosystems Imaging, USA).

**Immunofluorescence**

For cell immunofluorescence, cells were grown in glass coverslips. After indicated treatment, the cells were fixed with 4% paraformaldehyde, blocked with 5% bovine serum albumin in PBS and incubated with primary antibodies against α-SMA, CD86 or F4/80 overnight at 4°C. After washing, cells were incubated with secondary antibody for another 1 h at room temperature. The nuclei were stained with 4’, 6-diamidino-2-phenylindole (DAPI, Beyotime) and the images were captured using fluorescence microscopy (FV300, Olympus, Japan).

For tissue immunofluorescence, tissue sections were fixed with precooled acetone and incubated with the indicated primary antibodies overnight at 4°C. After washing with PBS, the sections were incubated with Alexa Fluor 488- or Alexa Fluor 594- or Alexa Fluor 650-conjugated secondary antibodies (Thermo Fisher Scientific) at room temperature for 1 h. The sections were stained with DAPI and the images were captured using Pannoramic SCAN II (3DHISTECH, Hungary).

**Flow Cytometry**

For flow cytometry-based analysis of monocyte-derived macrophage infiltration, liver tissues were cut into small pieces and digested in the solution containing 0.5 mg/ml collagenase type IV (Sigma) and 0.1 mg/ml DNase I (Sigma). Then the cell suspensions were filtered using a 100-mm cell strainer. After that, the cell suspensions were stained with relevant antibodies, and the contents of CD11B+Ly6C+ monocytes, CD11B+F4/80+CD163+ macrophages and CD11B+F4/80+CD86+ macrophages were detected by Fortessa flow cytometer (BD Biosciences, New Jersey, USA). And the mean fluorescence intensity (MFI) of Ly6C, CD163 and CD86 was calculated by FlowJo software (BD Biosciences, MD, USA).

**TCGA data collection**

The transcriptome data and corresponding clinical information of GBM and thyroid cancer cases with radiotherapy were downloaded from The Cancer Genome Atlas (TCGA) data portal (<https://tcga-data.nci.nih.gov/tcga/>). Based on the cut-points calculated by X-Tile Software[6], the cases were divided into high and low expression group of the indicated genes. The overall survival differences between high and low expression groups were evaluated by Kaplan-Meier survival analysis and log-rank test.

**Statistics**

The results represented means ± SEM. Statistical difference between two groups was compared by unpaired Student’s t test or the nonparametric Mann-Whitney U test. Statistical difference between multiple-group was compared by one-way or two-way analysis of variance (ANOVA). The significances of *p* value were shown as **p* < 0.05, ***p* < 0.01.

**Data availability**

The data that support the findings of this study are available from the corresponding author upon reasonable request.

References:

1. Chen Y, Wu Z, Yuan B, Dong Y, Zhang L,Zeng Z (2018) MicroRNA-146a-5p attenuates irradiation-induced and LPS-induced hepatic stellate cell activation and hepatocyte apoptosis through inhibition of TLR4 pathway. 9: 22. <https://doi.org/10.1038/s41419-017-0038-z>

2. Hou P P, Luo L J, Chen H Z, Chen Q T, Bian X L, Wu S F, Zhou J X, Zhao W X, Liu J M, Wang X M, Zhang Z Y, Yao L M, Chen Q, Zhou D,Wu Q (2020) Ectosomal PKM2 Promotes HCC by Inducing Macrophage Differentiation and Remodeling the Tumor Microenvironment. 78: 1192-1206.e1110. <https://doi.org/10.1016/j.molcel.2020.05.004>

3. Livak K J,Schmittgen T D (2001) Analysis of relative gene expression data using real-time quantitative PCR and the 2(-Delta Delta C(T)) Method. 25: 402-408. <https://doi.org/10.1006/meth.2001.1262>

4. Gagliardi M,Matarazzo M R (2016) RIP: RNA Immunoprecipitation. 1480: 73-86. <https://doi.org/10.1007/978-1-4939-6380-5_7>

5. Fang Y, Zhan Y, Xie Y, Du S, Chen Y, Zeng Z, Zhang Y, Chen K, Wang Y, Liang L, Ding Y,Wu D (2022) Integration of glucose and cardiolipin anabolism confers radiation resistance of HCC. 75: 1386-1401. <https://doi.org/10.1002/hep.32177>

6. Camp R L, Dolled-Filhart M,Rimm D L (2004) X-tile: a new bio-informatics tool for biomarker assessment and outcome-based cut-point optimization. 10: 7252-7259. <https://doi.org/10.1158/1078-0432.Ccr-04-0713>

**Table S2. Sequence of qRT-PCR primers**

| Gene | Forward | Reverse |
| --- | --- | --- |
| Human |  |  |
| ALKBH5 | ATGCACCCCGGTTGGAAAC | GACTTGCGCCAGTAGTTCTCA |
| TIRAP | ATGGCATCATCGACCTCCCT | GTCACTCGCATGTGTGGGT |
| BCL2 | GGTGGGGTCATGTGTGTGG | CGGTTCAGGTACTCAGTCATCC |
| ACTA2 | CTATGAGGGCTATGCCTTGCC | GCTCAGCAGTAGTAACGAAGGA |
| COL1A1 | CAGATCACGTCATCGCACAAC | GAGGGCCAAGACGAAGACATC |
| YTHDF2 | GGGGACAAGTGGGTCTCAAG | AGGGTGTCGCTGTGAAAGC |
| CD163 | TTTGTCAACTTGAGTCCCTTCAC | TCCCGCTACACTTGTTTTCAC |
| MRC1 | GGGTTGCTATCACTCTCTATGC | TTTCTTGTCTGTTGCCGTAGTT |
| IL10 | TCAAGGCGCATGTGAACTCC | GATGTCAAACTCACTCATGGCT |
| IL1B | TGGCAATGAGGATGACTTGT | TGGTGGTCGGAGATTCGTA |
| TNF | TCGTAGCAAACCACCAAGTG | AGATAGCAAATCGGCTGACG |
| NOS2 | AGGCCACCTCTATGTTTGCG | TGATAGCGCTTCTGGCTCTTG |
| CCL5 | CCAGCAGTCGTCTTTGTCAC | CTCTGGGTTGGCACACACTT |
| CXCL12 | ATTCTCAACACTCCAAACTGTGC | ACTTTAGCTTCGGGTCAATGC |
| CCL20 | TGCTGTACCAAGAGTTTGCTC | CGCACACAGACAACTTTTTCTTT |
| GAPDH | CTGGGCTACACTGAGCACC | AAGTGGTCGTTGAGGGCAATG |
| Mouse |  |  |
| *Alkbh5* | GCATACGGCCTCAGGACATTA | TTCCAATCGCGGTGCATCTAA |
| *Tirap* | CCTCCACTCCGTCCAAGAAG | TGAACCATCATAGAGGTGGCTTT |
| *Irak1* | TCCTCCACCAAGCAGTCAAG | AAAACCACCCTCTCCAATCCT |
| *Traf6* | AAAGCGAGAGATTCTTTCCCTG | ACTGGGGACAATTCACTAGAGC |
| *Bcl2* | GTCGCTACCGTCGTGACTTC | CAGACATGCACCTACCCAGC |
| *Acta2* | GGCACCACTGAACCCTAAGG | ACAATACCAGTTGTACGTCCAGA |
| *Col1a1* | TGACTGGAAGAGCGGAGAGT | GACGGCTGAGTAGGGAACAC |
| *Arg1* | CTCCAAGCCAAAGTCCTTAGAG | AGGAGCTGTCATTAGGGACATC |
| *Mrc1* | GCTTCCGTCACCCTGTATGC | TCATCCGTGGTTCCATAGACC |
| *Il10* | GCTGGACAACATACTGCTAACC | ATTTCCGATAAGGCTTGGCAA |
| *Il1b* | GAAATGCCACCTTTTGACAGTG | TGGATGCTCTCATCAGGACAG |
| *Tnf* | TCGTAGCAAACCACCAAGTG | AGATAGCAAATCGGCTGACG |
| *Nos2* | GTTCTCAGCCCAACAATACAAGA | GTGGACGGGTCGATGTCAC |
| *Ccl5* | TTTGCCTACCTCTCCCTCG | CGACTGCAAGATTGGAGCACT |
| *Ccl20* | GCCTCTCGTACATACAGACGC | CCAGTTCTGCTTTGGATCAGC |
| *Gapdh* | TGTGTCCGTCGTGGATCTGA | TTCGTGTTGAAGTCGCAGGAG |

**Table S3. Antibodies**

| Name | Supplier | Cat No. | Dilution (WB) | Dilution (IHC/IF) |
| --- | --- | --- | --- | --- |
| ALKBH5 | Proteintech | 16837-1-AP | 1:4000 | 1:200 |
| TIRAP | Abcam | ab17218 | 1:1000 | 1:500 |
| IRAK1 | Proteintech | 10478-2-AP | 1:1000 | 1:600 |
| TRAF6 | Abcam | ab33915 | 1:1000 | 1:300 |
| α-SMA | Abcam | ab32575 | 1:5000 | 1:1000 |
| NF-κB p65 | CST | 8242 | 1:1000 |  |
| phospho-NF-κB p65 (Ser536) | CST | 3033 | 1:1000 |  |
| SAPK/JNK | CST | 9252 | 1:1000 |  |
| phospho-SAPK/JNK (Thr183/Tyr185) | CST | 4668 | 1:1000 |  |
| Smad2 | CST | 5339 | 1:1000 |  |
| phospho-Smad2 (Ser465/467) | CST | 3101 | 1:1000 |  |
| Bcl-2 | CST | 3498 | 1:1000 |  |
| cleaved caspase-3 (Asp175) | CST | 9661 | 1:1000 |  |
| Collagen1 | Abcam | ab260043 | 1:1000 |  |
| F4/80 | Abcam | ab111101 |  | 1:100 |
| GAPDH | CST | 2118 | 1:1000 |  |
| IBA1 | Proteintech | 10904-1-AP |  | 1:200 |
| CLEC4F | R&D Systems | AF2784 |  | 1:50 |
| CD68 | Abcam | ab201340 |  | 1:100 |
| AKT | Proteintech | 10176-2-AP | 1:1000 |  |
| phospho-AKT | Proteintech | 66444-1-Ig | 1:2000 |  |
| STAT3 | Proteintech | 10253-2-AP | 1:1000 |  |
| phospho-STAT3 | Immunoway | YP0250 | 1:1000 |  |
| ERK | Proteintech | 16443-1-AP | 1:2000 |  |
| phospho-ERK | Immunoway | YP0101 | 1:1000 |  |
| CCR5 | Immunoway | YT0936 |  | 1:200 |
| CD86 | CST | 19589 |  | 1:200 |
| CD163 | Abcam | ab182422 |  | 1:200 |

**Table S1. Patient information**

| Case | Specimen | Age | Sex | RT area | RT dose | RT type | RILI | Tumor response |
| --- | --- | --- | --- | --- | --- | --- | --- | --- |
| 1 | before RT | 32 | M | PVTT | 42Gy/7F | IMRT | YES | NO |
| 2 | before RT | 45 | M | intrahepatic tumor | 48Gy/8F | IMRT | YES | NO |
| 3 | before RT | 45 | M | intrahepatic tumor  & PVTT | 60Gy/30F | IMRT | YES | NO |
| 4 | before RT | 42 | M | intrahepatic tumor | 56Gy/7F | IMRT | YES | NO |
| 5 | before RT | 36 | M | intrahepatic tumor | 48Gy/8F | IMRT | YES | NO |
| 6 | Post RT | 50 | M | intrahepatic tumor | 48Gy/6F | IMRT | YES | NO |
| 7 | Post RT | 50 | M | intrahepatic tumor  & PVTT | 54Gy/18F | IMRT | YES | NO |
| 8 | before RT | 46 | M | intrahepatic tumor | 54Gy/18F | IMRT | NO | YES |
| 9 | before RT | 48 | M | intrahepatic tumor | 50Gy/10F | IMRT | NO | YES |
| 10 | before RT | 49 | M | intrahepatic tumor | 60Gy/28F | IMRT | NO | YES |
| 11 | before RT | 46 | M | intrahepatic tumor | 62.5Gy/25F | IMRT | NO | YES |
| 12 | before RT | 47 | M | intrahepatic tumor | 60Gy/20F | IMRT | NO | YES |
| 13 | before RT | 26 | M | intrahepatic tumor | 60Gy/25F | IMRT | NO | YES |
| 14 | before RT | 47 | M | PVTT | 60Gy/28F | IMRT | NO | YES |
| 15 | before RT | 66 | M | intrahepatic tumor | 50Gy/10F | IMRT | NO | YES |
| 16 | before RT | 45 | F | intrahepatic tumor | 50Gy/10F | IMRT | NO | YES |
| 17 | before RT | 60 | M | intrahepatic tumor  & PVTT | 62.5Gy/25F | IMRT | NO | YES |

Note: RT, radiotherapy; PVTT, portal vein tumor thrombus; IMRT, intensity-modulated radiation therapy

**Supplementary Figures and Legends:**


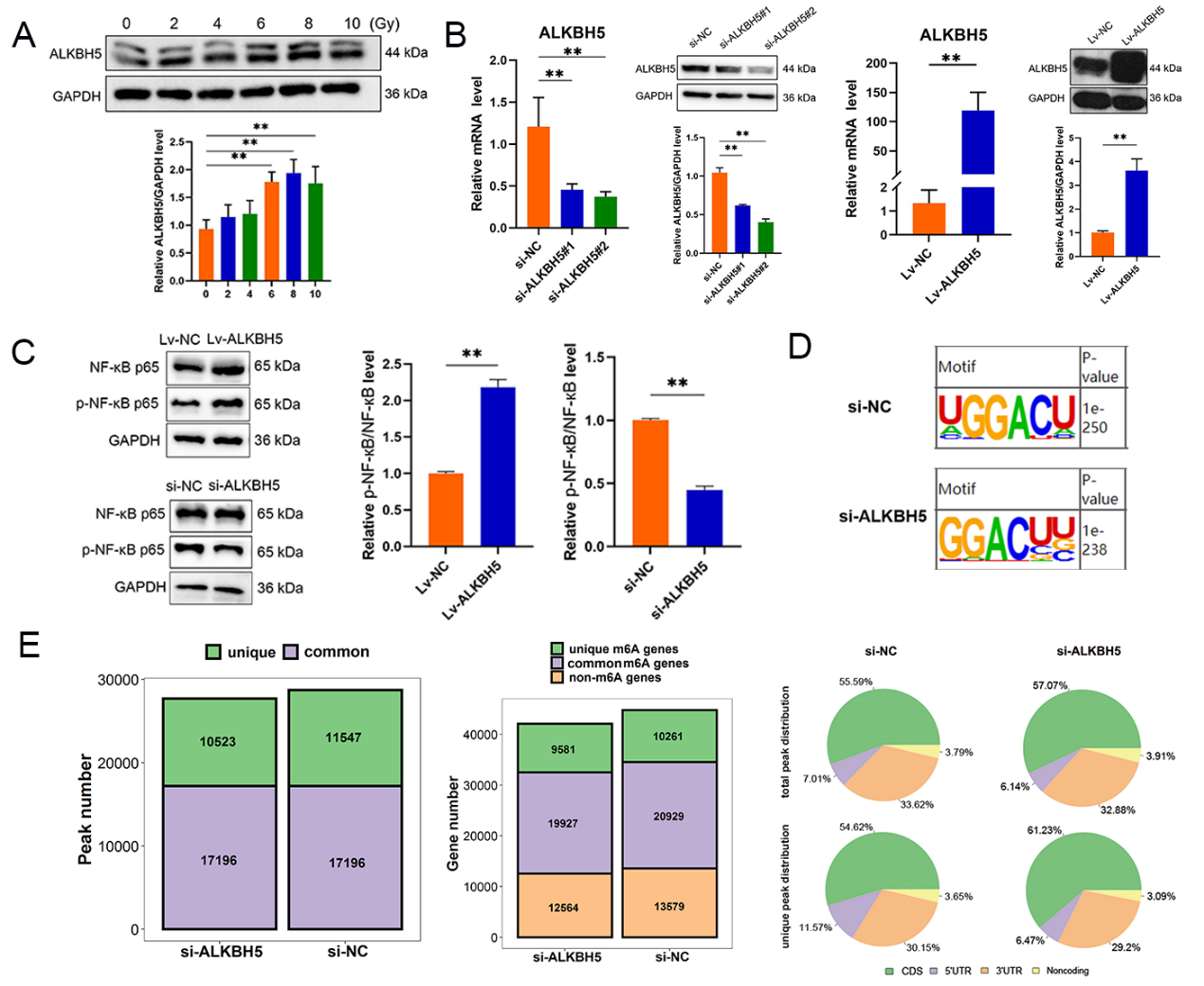


**Figure S1 Upregulation of ALKBH5 in HSC promotes the activation of NF-κB**

(A) Western blot analysis of ALKBH5 in LX2 cells with various doses of irradiation. ***p* < 0.01.

(B) mRNA and Western blot analysis of ALKBH5 in LX2 cells treated with different ALKBH5 siRNAs or ALKBH5-overexpressing lentivirus. ***p* < 0.01. si-ALKBH5, ALKBH5 siRNA; si-NC, siRNA negative control; Lv-ALKBH5, ALKBH5-overexpressing lentivirus; Lv-NC，ALKBH5-overexpressing lentivirus negative control.

(C) Western blot analysis of NF-κB phosphorylation in IR-LX2 cells with knockdown or overexpression of ALKBH5. ***p* < 0.01.

(D) The m^6^A consensus motif of IR-LX2 cells.

(E) The number of m^6^A peaks (left panel), m^6^A-modified transcripts (middle panel) and distribution of total and unique m^6^A peaks (right panel) in IR-LX2 cells transfected with si-NC or si-ALKBH5.


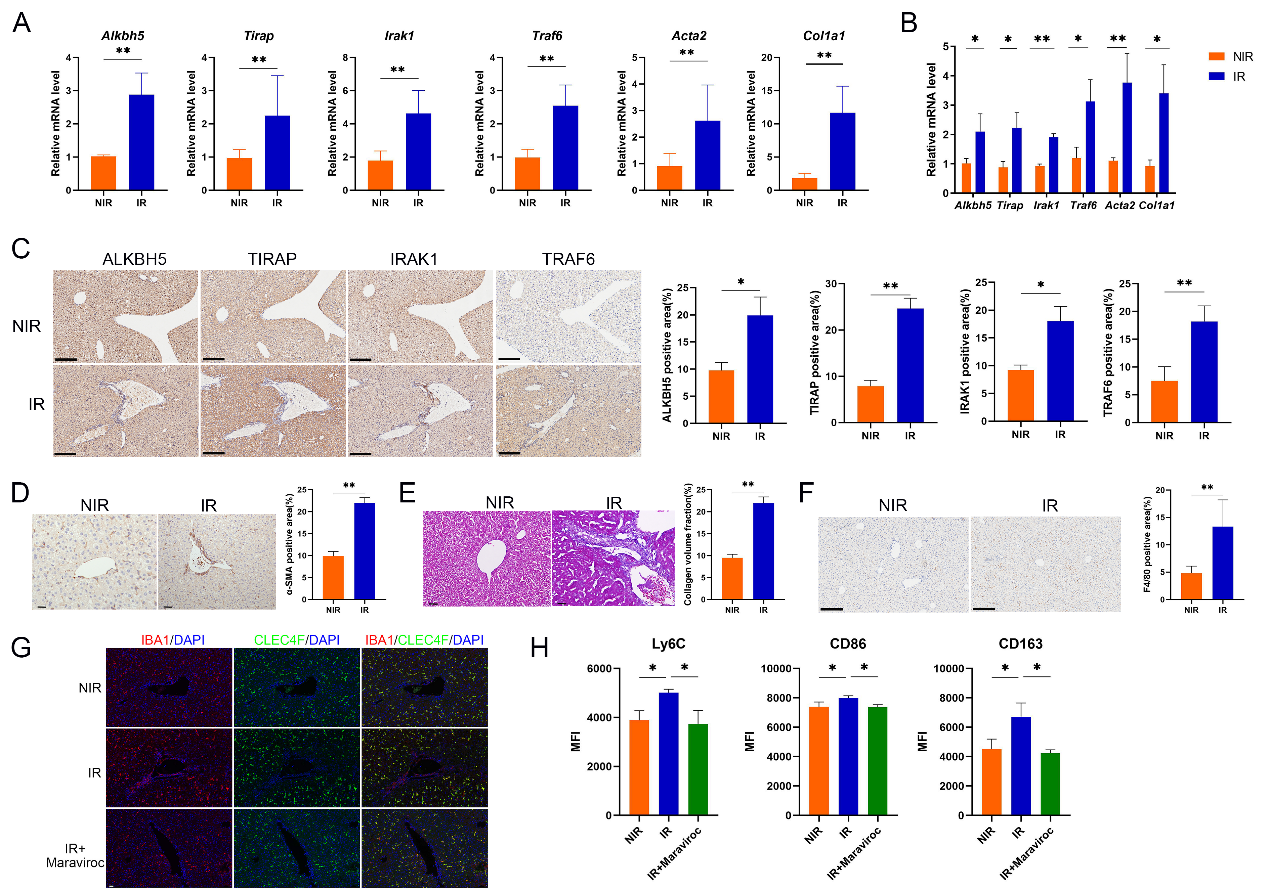


**Figure S2 Irradiation promotes HSC activation and monocyte infiltration in liver of mice**

(A-B) mRNA level of Alkbh5, Tirap, Irak1, Traf6, Acta2 and Col1a1 in liver(A) and HSCs(B) from NIR and IR mice. ***p* < 0.01.

(C) IHC staining of ALKBH5, TIRAP, IRAK1 and TRAF6 in liver from NIR and IR mice. ***p* < 0.01; **p* < 0.05. Scale bar: 200 μm.

(D) IHC staining of α-SMA in liver from NIR and IR mice. ***p* < 0.01. Scale bar: 50 μm.

(E) Masson staining in liver from NIR and IR mice. ***p* < 0.01. Scale bar: 50 μm.

(F) IHC staining of F4/80 in liver from NIR and IR mice. ***p* < 0.01. Scale bar: 200 μm.

(G) IBA1 and CLEC4F fluorescence in liver from NIR and IR mice. Scale bar: 20 μm.

(H) Flow cytometry detection of the expression levels of monocyte marker Ly6C, M1 marker CD86 and M2 marker CD163 in liver tissues from indicated mice. MFI, mean fluorescence intensity. **p* < 0.05.


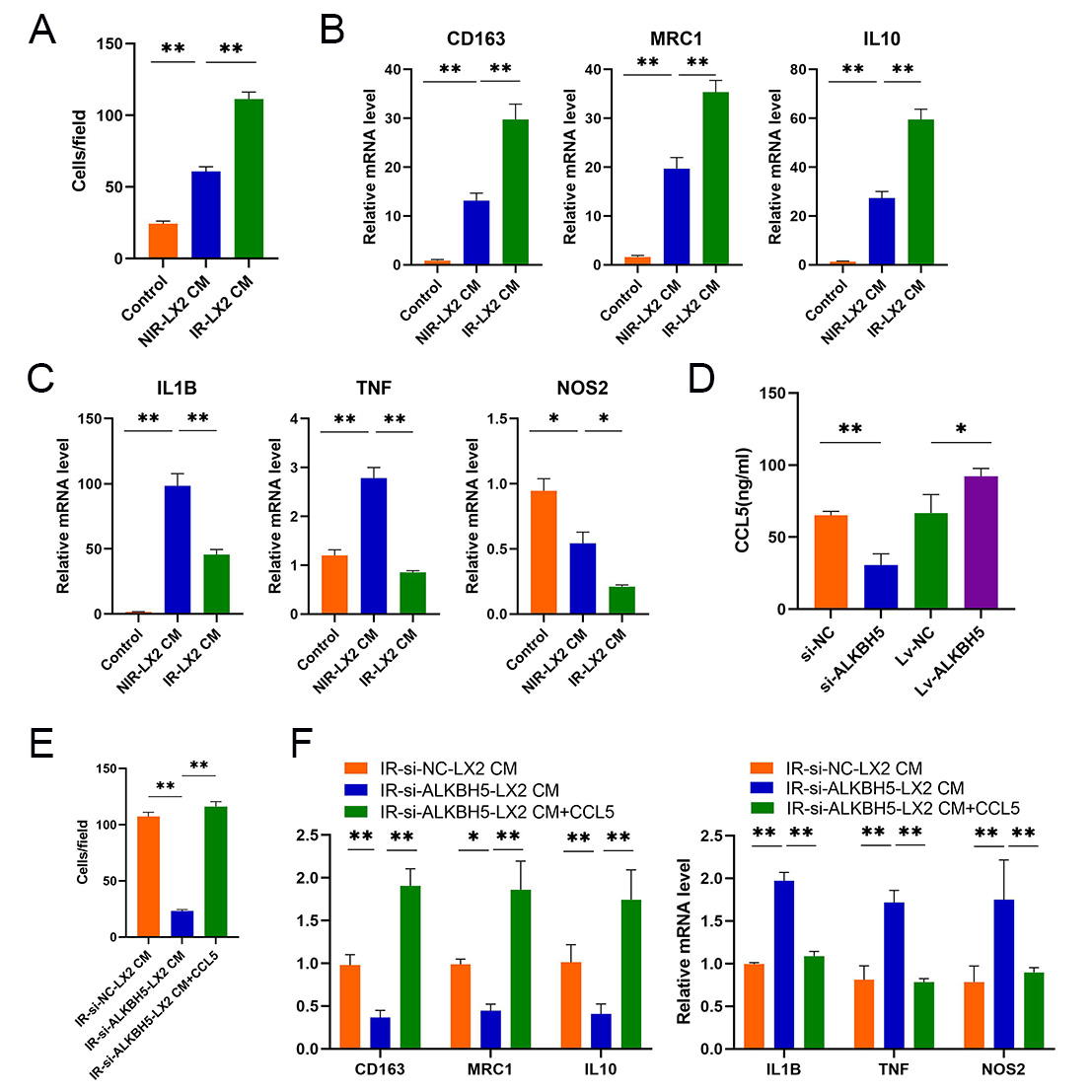


**Figure S3 IR-LX2 cells promote THP-1 cells recruitment and polarization**

(A) Cell migration of THP-1 after co-culturing with NIR-LX2 CM and IR-LX2 CM. ***p* < 0.01.

(B) mRNA level of M2 markers (CD163, MRC1 and IL10) in THP-1 cells after co-culturing with NIR-LX2 CM and IR-LX2 CM. ***p* < 0.01.

(C) mRNA level of M1 markers (IL1B, TNF and NOS2) in THP-1 cells after co-culturing with NIR-LX2 CM and IR-LX2 CM. ***p* < 0.01.

(D) ELISA detection of CCL5 in CM from IR-LX2 with altered ALKBH5 expression. ***p* < 0.01; **p* < 0.05.

(E) Cell migration of THP-1 after co-culturing with control or ALKBH5-silenced IR-LX2 CM with or without CCL5(20 ng/ml) treatment. ***p* < 0.01.

(F) mRNA level of polarization markers in THP-1 cells after co-culturing with control or ALKBH5-silenced IR-LX2 CM with or without CCL5(20 ng/ml) treatment. ***p* < 0.01; **p* < 0.05.


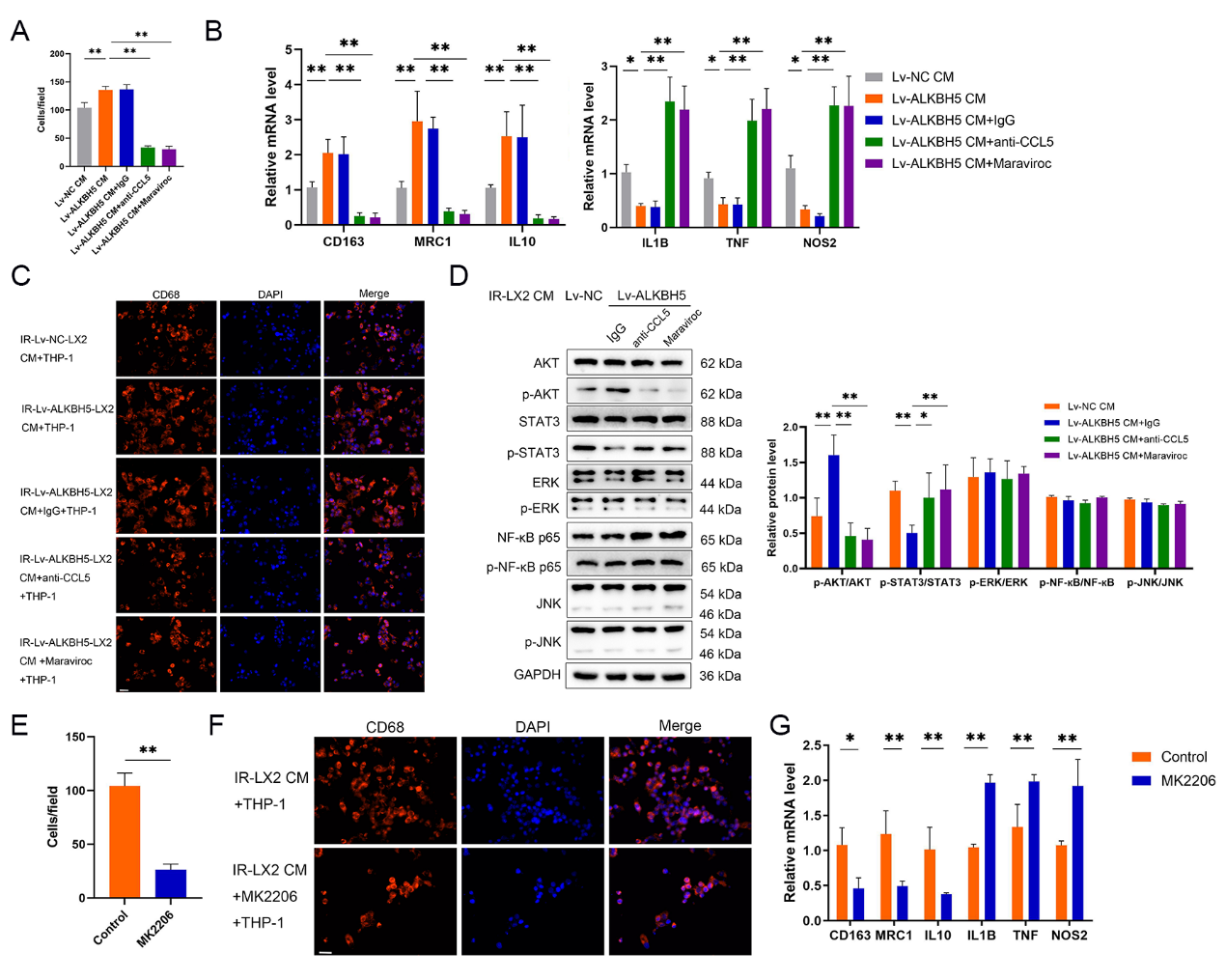


**Figure S4 Overexpression of ALKBH5 in IR-LX2 cells promotes THP-1 cells recruitment and polarization**

(A-D) THP-1 cells co-cultured with control or ALKBH5 overexpressing IR-LX2 CM in the presence of control IgG, α-CCL5(10 μg/ml) or maraviroc (5 μM). The cell migration(A), mRNA level of polarization markers(B), CD68 fluorescence(C), Western blot analysis of related proteins(D) in THP-1 cells were detected. ***p* < 0.01; **p* < 0.05. Scale bar: 50 μm.

(E-F) Control or MK2206(500 nM) pretreated THP-1 cells co-cultured with IR-LX2 CM. The cell migration(E), mRNA level of polarization markers(F) and CD68 fluorescence(G) in THP-1 cells were detected. ***p* < 0.01; **p* < 0.05. Scale bar: 50 μm.


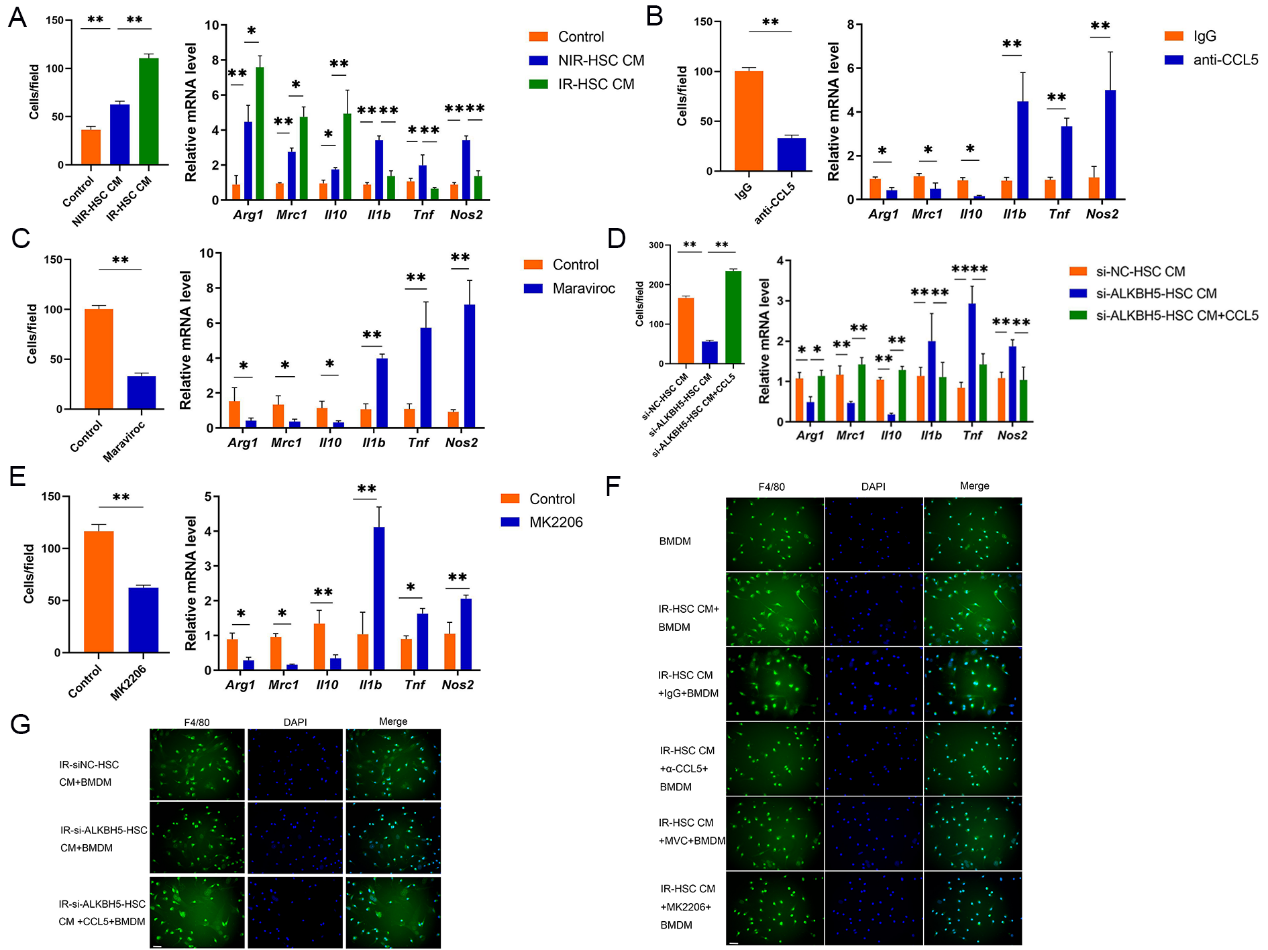


**Figure S5 Irradiated mouse HSCs promote BMDM recruitment and polarization through CCL5-CCR5 axis**

(A) Cell migration (left panel) and polarization (right panel) of bone marrow derived monocyte (BMDM) after co-culturing with nonirradiated mouse HSC (NIR-HSC) CM or irradiated mouse HSC (IR-HSC) CM. ***p* < 0.01; **p* < 0.05.

(B) Cell migration (left panel) and polarization (right panel) of BMDM after co-culturing with IR-HSC CM in the presence of anti-CCL5 neutralizing antibody (α-CCL5, 10 μg/ml) or control IgG. ***p* < 0.01; **p* < 0.05.

(C) Cell migration (left panel) and polarization (right panel) of maraviroc (5 μM) pretreated BMDM after co-culturing with IR-HSC CM. ***p* < 0.01; **p* < 0.05.

(D) Cell migration (left panel) and polarization (right panel) of BMDM after co-culturing with control or ALKBH5-silenced IR-HSC CM with or without CCL5 (20 ng/ml) treatment. ***p* < 0.01; **p* < 0.05.

(E) Cell migration (left panel) and polarization (right panel) of MK2206 (500 nM) pretreated BMDM after co-culturing with IR-HSC CM. ***p* < 0.01; **p* < 0.05.

(F) CD68 fluorescence in BMDM after co-culturing with IR-HSC CM in the presence of α-CCL5 (10 μg/ml), maraviroc (5 μM) or MK2206 (500 nM). Scale bar: 50 μm.

(G) CD68 fluorescence in BMDM after co-culturing with control or ALKBH5-silenced IR-HSC CM with or without CCL5 (20 ng/ml) treatment. Scale bar: 50 μm.


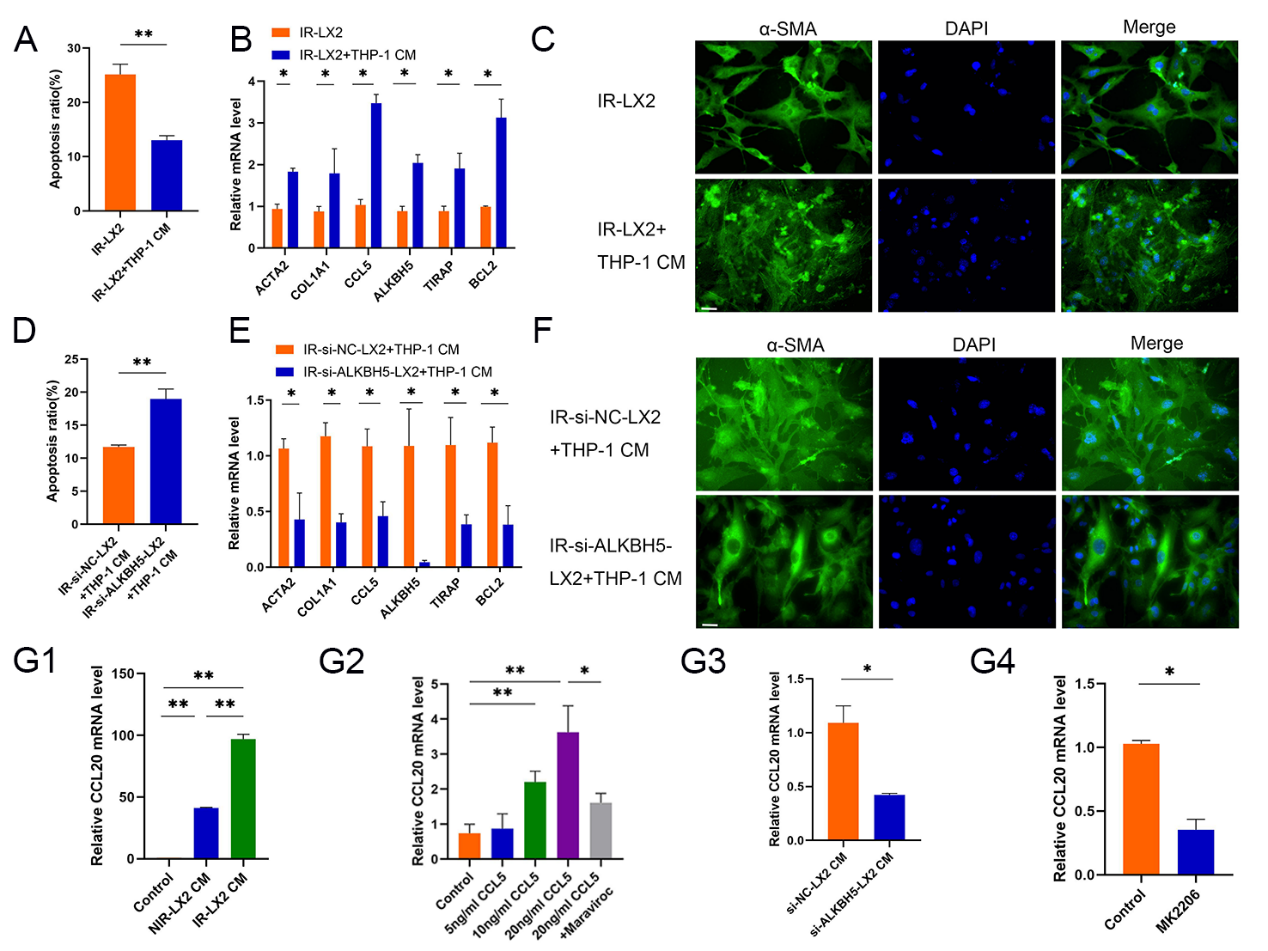


**Figure S6 IR-LX2 educated THP-1 cells up-regulate the expression of ALKBH5 in IR-LX2**

(A) Apoptotic cell detection of IR-LX2 cells after co-culturing with CM from IR-LX2 CM-stimulated THP-1 (THP-1 CM). ***p* < 0.01.

(B) mRNA level of fibrotic markers (ACTA2, COL1A1), chemokine (CCL5) and ALKBH5 in IR-LX2 cells after co-culturing with THP-1 CM. **p* < 0.05.

(C) α-SMA fluorescence in IR-LX2 cells after co-culturing with THP-1 CM. Scale bar: 50 μm.

(D) Apoptotic cell detection of control or ALKBH5-silenced IR-LX2 cells after co-culturing with THP-1 CM. ***p* < 0.01.

(E) mRNA level of fibrotic markers (ACTA2, COL1A1), chemokine (CCL5) and ALKBH5 in control or ALKBH5-silenced IR-LX2 cells after co-culturing with THP-1 CM. **p* < 0.05.

(F) α-SMA fluorescence in control or ALKBH5-silenced IR-LX2 cells after co-culturing with THP-1 CM. Scale bar: 50 μm.

(G) mRNA level of CCL20 in THP-1 cells as indicated treatment. (G1) THP-1 cells co-cultured with NIR or IR-LX2 CM. (G2) THP-1 cells were treated with various doses of CCL5 with or without MVC (5 μM). (G3) THP-1 cells co-cultured with control or ALKBH5-silenced IR-LX2 CM. (G4) Control or MK2206 (500 nM) pretreated THP-1 cells co-cultured with IR-LX2 CM. ***p* < 0.01; **p* < 0.05.


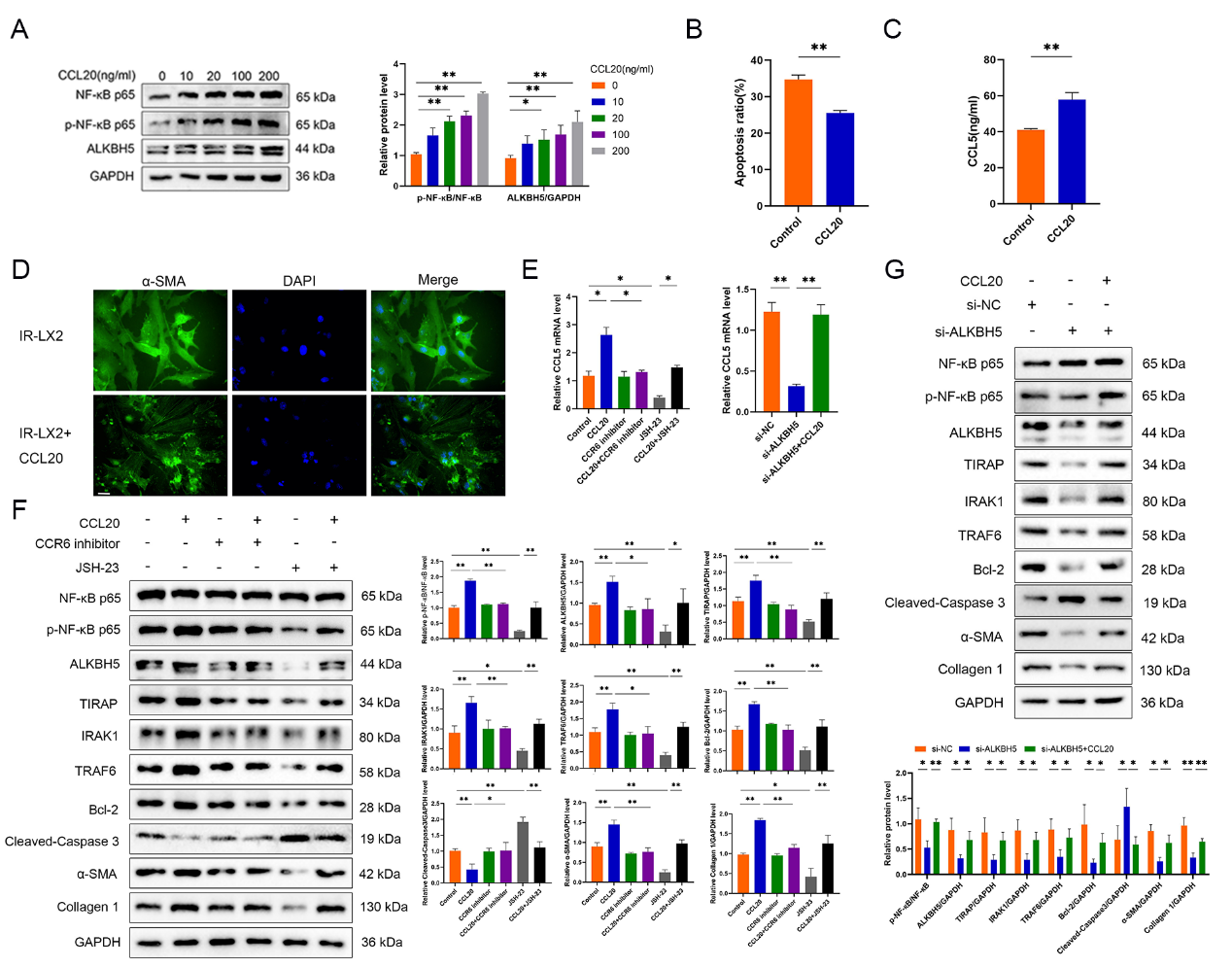


**Figure S7 CCL20 promotes cell activation and CCL5 production of IR-LX2 cells through upregulation of ALKBH5**

(A) Western blot analysis of NF-κB phosphorylation and ALKBH5 in IR-LX2 cells with various doses of CCL20 stimulation. ***p* < 0.01; **p* < 0.05.

(B) Apoptotic cell detection of IR-LX2 cells treated with or without CCL20 (200 ng/ml). ***p* < 0.01.

(C) ELISA detection of CCL5 secretion from IR-LX2 cells treated with or without CCL20 (200 ng/ml). ***p* < 0.01.

(D) α-SMA fluorescence in IR-LX2 cells treated with or without CCL20 (200 ng/ml). Scale bar: 50 μm.

(E) mRNA level of CCL5 in IR-LX2 cells treated with CCL20 (200 ng/ml), CCR6 inhibitor (20 μM), JSH-23 (50 μM) or in combination. ***p* < 0.01; **p* < 0.05.

(F) Western blot analysis of related proteins in IR-LX2 cells treated with CCL20 (200 ng/ml), CCR6 inhibitor (20 μM), JSH-23 (50 μM) or in combination. ***p* < 0.01; **p* < 0.05.

(G) Western blot analysis of related proteins in control or ALKBH5-silenced IR-LX2 cells with or without CCL20(200 ng/ml) treatment. ***p* < 0.01; **p* < 0.05.


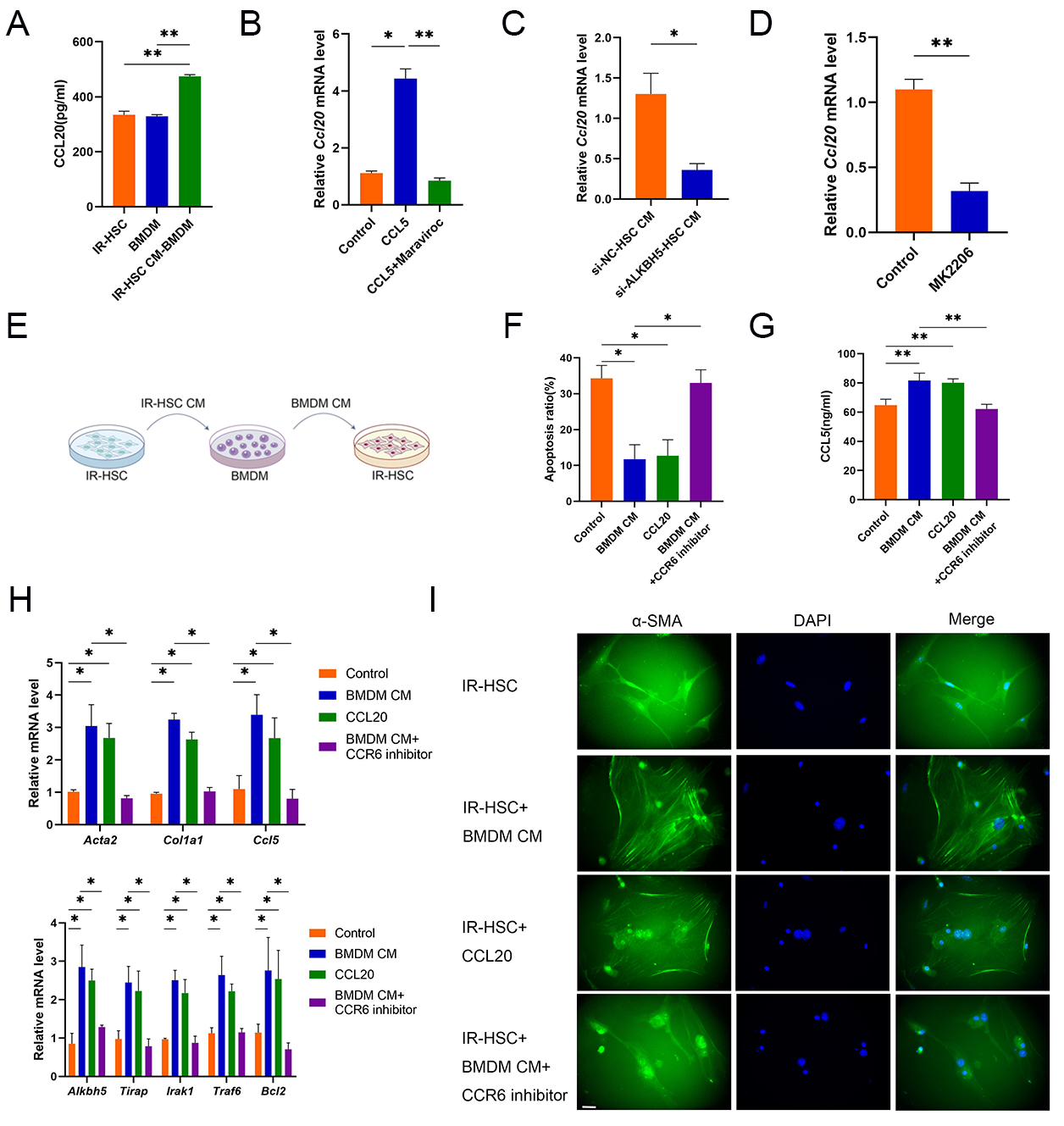


**Figure S8 Irradiated mouse HSC educated BMDM promotes cell activation and CCL5 production of HSC through CCL20**

(A) ELISA detection of CCL20 secretion from irradiated mouse HSC (IR-HSC), BMDM and IR-HSC CM stimulated BMDM. ***p* < 0.01.

(B) mRNA level of *Ccl20* in BMDM treated with CCL5 (20 ng/ml) or combined with MVC (5 μM). ***p* < 0.01; **p* < 0.05.

(C) mRNA level of *Ccl20* in BMDM after co-culturing with control or ALKBH5-silenced IR-HSC CM. **p* < 0.05.

(D) mRNA level of *Ccl20* in control or MK2206 (500 nM) pretreated BMDM after co-culturing with IR-HSC CM. ***p* < 0.01.

(E) Schematic overview of the coculture. BMDM CM was collected from IR-HSC CM stimulated BMDM and then co-cultured with IR-HSC.

(F) Apoptotic cell detection of IR-HSC after co-culturing with CCL20 (200 ng/ml), BMDM CM, CCR6 inhibitor (20 μM) or in combination. **p* < 0.05.

(G) ELISA detection of CCL5 secretion from IR-HSC after co-culturing with CCL20 (200 ng/ml), BMDM CM, CCR6 inhibitor (20 μM) or in combination. ***p* < 0.01.

(H) mRNA level of fibrotic markers (*Acta2, Col1a1*), chemokine (*Ccl5*) and *Alkbh5* downstream genes in IR-HSC after co-culturing with CCL20 (200 ng/ml), BMDM CM, CCR6 inhibitor (20 μM) or in combination. **p* < 0.05.

(I) α-SMA fluorescence in IR-HSC after co-culturing with CCL20 (200 ng/ml), BMDM CM, CCR6 inhibitor (20 μM) or in combination. Scale bar: 50 μm.


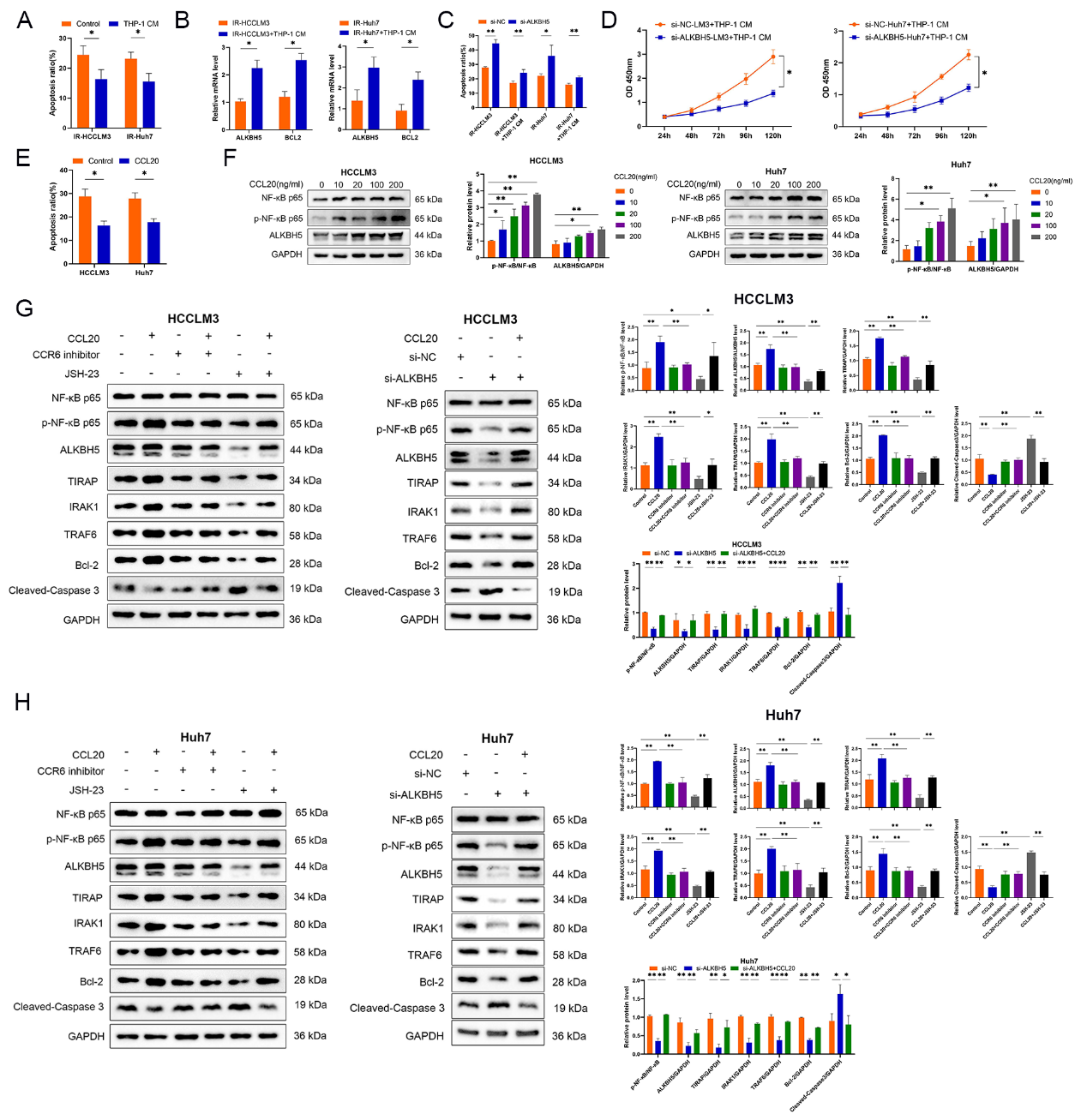


**Figure S9 CCL20 reduces radiosensitivity of HCC cells through upregulation of ALKBH5**

(A) Apoptotic cell detection of irradiated HCC (IR-HCC) cells after co-culturing with CM from IR-LX2 CM-stimulated THP-1 (THP-1 CM). **p* < 0.05.

(B) mRNA level of ALKBH5 and BCL2 in IR-HCC cells after co-culturing with THP-1 CM. **p* < 0.05.

(C) Apoptotic cell detection of control or ALKBH5-silenced IR-HCC cells in the presence or absence of THP-1 CM. ***p* < 0.01; **p* < 0.05.

(D) CCK8 proliferation assay of control or ALKBH5-silenced IR-HCC cells co-cultured with THP-1 CM. **p* < 0.05.

(E) Apoptotic cell detection of IR-HCC cells treated with or without CCL20 (200 ng/ml). ***p* < 0.01; **p* < 0.05.

(F) Western blot analysis of NF-κB phosphorylation and ALKBH5 in IR-HCC cells with various doses of CCL20 stimulation. **p* < 0.05.

(G-H) Western blot analysis of related proteins in IR-HCC cells treated with CCL20 (200 ng/ml), CCR6 inhibitor (20 μM), JSH-23 (50 μM) or in combination (G-H left panel). Western blot analysis of related proteins in control or ALKBH5-silenced IR-HCC cells with or without CCL20 (200 ng/ml) treatment (G-H middle panel). Relative quantification for the protein bands (G-H right panel). ***p* < 0.01; **p* < 0.05.

**
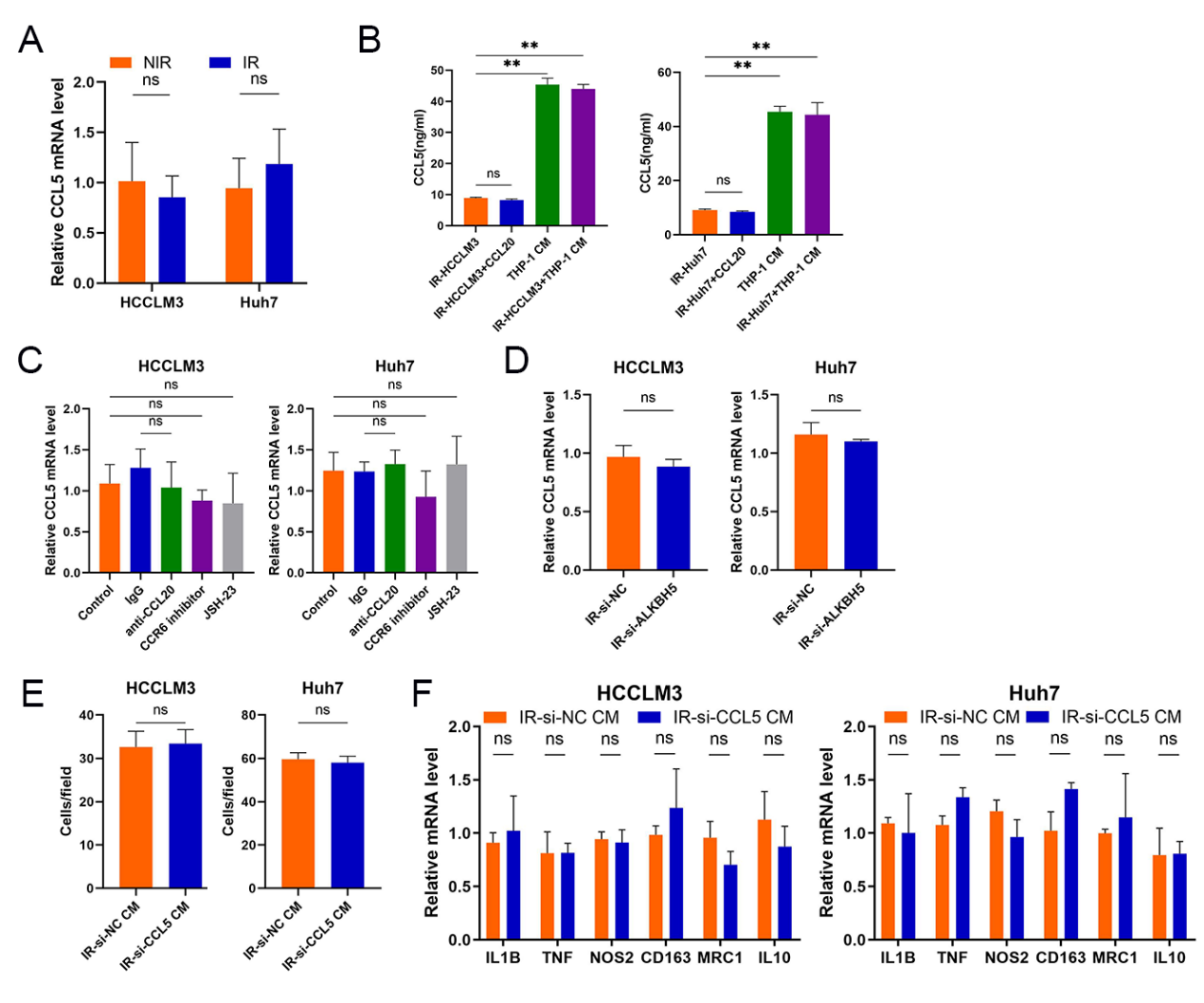
**

**Figure S10 Expression of CCL5 in HCC cells has no effect on the migration and polarization of monocytes**

(A) mRNA level of CCL5 in NIR- or IR-HCC cells.

(B) ELISA detection of CCL5 secretion from IR-HCC cells, CCL20 (200 ng/ml) stimulated IR-HCC cells, IR-LX2 CM-stimulated TPH-1 cells (THP-1 CM) or THP-1 CM stimulated IR-HCC cells. ***p* < 0.01.

(C) mRNA level of CCL5 in IR-HCC cells treated with CCL20 (200 ng/ml), CCR6 inhibitor (20 μM), JSH-23 (50 μM) or in combination.

(D) mRNA level of CCL5 in control or ALKBH5-silenced IR-HCC cells.

(E) Cell migration of THP-1 cells after co-culturing with control or CCL5-silenced IR-HCC CM.

(F) mRNA level of polarization markers in THP-1 cells after co-culturing with control or CCL5-silenced IR-HCC CM.
